# The value of understanding feedbacks from ecosystem functions to species for managing ecosystems

**DOI:** 10.1038/s41467-019-11890-7

**Published:** 2019-08-29

**Authors:** Hui Xiao, Eve McDonald-Madden, Régis Sabbadin, Nathalie Peyrard, Laura E. Dee, Iadine Chadès

**Affiliations:** 1 Centre for Biodiversity and Conservation Science, School of Earth and Environmental Science, University of Queensland, St Lucia, 4072 Australia; 2 CSIRO, EcoSciences Precinct, 41 Boggo Road, Dutton Park, QLD 4102 Australia; 3 ARC Centre for Excellence for Environmental Decisions, University of Queensland, St Lucia, 4072 Australia; 4 MIAT, UR 875, Université de Toulouse, INRA, Castanet-Tolosan, F-31320 France; 5 Department of Fisheries, Wildlife, and Conservation Biology, University of Minnesota, Twin Cities, St. Paul, MN 55108 USA; 6 Department of Ecology and Evolutionary Biology, University of Colorado at Boulder, Boulder, CO 80309 USA

**Keywords:** Ecosystem ecology, Ecological modelling, Ecological networks

## Abstract

Ecological systems are made up of complex and often unknown interactions and feedbacks. Uncovering these interactions and feedbacks among species, ecosystem functions, and ecosystem services is challenging, costly, and time-consuming. Here, we ask: for which ecosystem features does resolving the uncertainty about the feedbacks from ecosystem function to species improve management outcomes? We develop a dynamic value of information analysis for risk-neutral and risk-prone managers on motif ecosystems and explore the influence of five ecological features. We find that learning the feedbacks from ecosystem function to species does not improve management outcomes for maximising biodiversity, yet learning which species benefit from an ecosystem function improves management outcomes for ecosystem services by up to 25% for risk-neutral managers and 231% for risk-prone managers. Our general approach provides useful guidance for managers and researchers on when learning feedbacks from ecosystem function to species can improve management outcomes for multiple conservation objectives.

## Abstract (web summary)

Value of information analyses are a promising approach to decision-making in conservation. Here the authors develop a dynamic approach to show that knowing which species benefit from an ecosystem function improves ecosystem service and biodiversity management, particularly for risk-prone managers.

## Introduction

Ecosystems are experiencing dramatic degradation worldwide, making optimal management a pressing topic for both science and practice given limited resources for conservation^1–4^. At the simplest level, ecosystems are a tangled web of plants and animals connected by feeding links, but further interactions exist between species, ecosystem function, and the services they provide^5^. Ecosystem functions not only contribute to the production of ecosystem services (e.g., nutrient cycling that supports soil fertility and boosts crop production) but also support the survival of species in an ecosystem^6,7^. We refer to this critical support that ecosystem functions provide for species survival as ‘ecosystem function-species feedbacks.’ Ecosystem function-species feedbacks have been observed in numerous empirical studies^8^ yet rarely considered for guiding ecosystem management. For instance, coral reefs provide nutrient cycling functions that can support both fisheries species (i.e., an ecosystem service) and shark populations (i.e., biodiversity)^9–11^. Similarly, pollination functions provided by birds and insects are critical to both crop productions and native plants within the community^12^. Consequently, species extinctions that degrade ecosystem functions can reduce the quantity or quality of ecosystem services while also threatening the survival of other species in the community that benefit from ecosystem function-species feedbacks. In our above examples, reduced nutrient cycling function could threaten both the service provision (i.e., coral reef-dependent fisheries) and biodiversity (i.e., shark populations)^13,14^. Similarly, decreased pollination from local pollinator extirpations could affect crop production and pollinator-dependent native plants^15,16^. Learning about these feedback links from ecosystem function to species could be important to inform decisions on species management priority to maximise biodiversity or ecosystem services. However, to the best of our knowledge, consideration of how this information may impact species management priorities is non-existent^3,17–21^.

In the past few decades, a growing suite of research on ecological interactions and networks has considered feedbacks between ecosystem functions and species. Those feedbacks have been represented as non-trophic links between species, such as mutualistic interactions that facilitate biodiversity maintenance^6,7,22–26^. The majority of this work, however, focuses on the stability of food webs to perturbations, rather than the management of these systems in light of the feedbacks between ecosystem functions and species^5^. Other studies investigating ecosystem function—species relationship using bio-economic models have focused on specific communities, such as mangrove-fishery ecosystems, pollinator-plant communities, or forests, for ecosystem service management. Yet those studies are restricted to two or three species where the exact ecosystem function—species relationships are assumed to be known^27,28^, which is rarely the case. The smaller set of studies that consider and model the potential importance of feedbacks for management do not quantify the benefits of collecting this information for management outcomes^29^. For instance, fishery management studies have acknowledged that ecosystem functions benefit both ecosystem services and endangered species’ recovery^30–32^. However, these studies perform scenario-based analysis that do not assess whether collecting information on feedbacks from ecosystem functions to species improve biodiversity or ecosystem services outcomes^33^.

In practice, collecting information about feedbacks, i.e., the set and strength of links from an ecosystem function to different species in the food web, is challenging, costly, and time consuming. However, knowing this information could potentially greatly improve management outcomes. Because ecosystem functions support both service provision and species survival in ecosystems, such information about these links could improve management by aiding the identification of win–win management strategies for maximising biodiversity and ecosystem services benefits^4,31,34^. Yet, determining whether collecting feedback information will improve species management outcomes and under what ecological conditions remains an open question^24^.

**Fig. 1:**
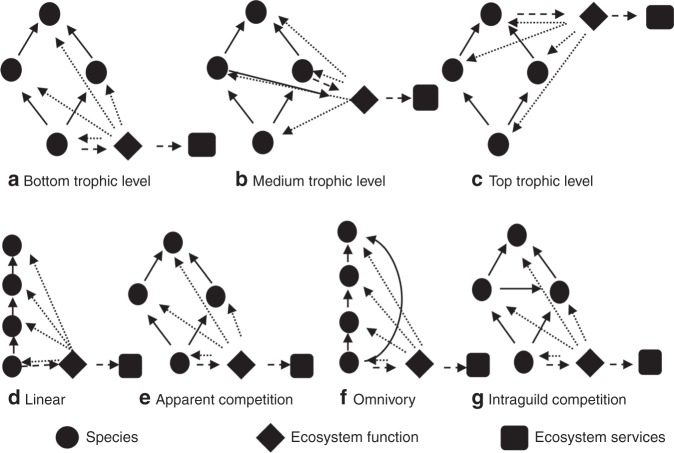
Examples of ecosystem network structures. **a**–**c** with different trophic levels performing an ecosystem function. **d**–**g** for different network motifs. For the same motif, an ecosystem function could be performed by **a** the bottom trophic level, **b** a medium trophic level, or **c** a top predator. Similarly, for the same species-function relationship (e.g., the bottom trophic level provides the ecosystem function), motifs could be **d** linear, **e** apparent competition, **f** omnivory, or **g** intraguild competition. The solid arrows represent known interactions—here a feeding relationship between a predator and its prey. The dashed arrows represent links from a species to the ecosystem function (diamonds) and services (rectangles) that it provides. The dotted arrows represent unknown interactions, representing feedbacks from an ecosystem function to species (e.g., pollination supports the reproduction of plants). This figure represents one of the 16 potential feedback structures—the ecosystem function benefits all species in the motif (see Supplementary Fig. 8 subplot (p))

We develop an approach to evaluate when resolving uncertainty about feedbacks from ecosystem function to species will improve management outcomes for maximising biodiversity and ecosystem services benefits. We combine value of information analysis—an approach for quantifying how much management outcomes could be improved if a decision-maker could resolve uncertainties^35^, and stochastic dynamic programming—an optimisation approach that has been widely applied on sequential decision-making problems^36–38^. To model the complex ecosystem dynamics we use four species motifs, which are common species interaction sub-structures identified from large empirical food webs^39^. Motifs have served as a convenient tool for studying system stability and evolution^40–42^ and have been utilised to compute management priorities for threatened and pest species, and disease networks^43^. To evaluate the feedbacks with ecosystem services we connect each motif with one major function that contributes to a key ecosystem service in the system (Fig. 1 and Methods). Using this model we investigate how different ecological features, such as the motif, the feedback strength and the trophic level of the species providing the ecosystem function (Fig. 1), drive the management benefit of this information. We estimate the value of having the feedback information using the relative Expected Value of Perfect Information (EVPI), which is obtained by dividing the absolute EVPI by the total number of species or the total value of the ecosystem services depending on the management objective^44,45^ (see Methods and main steps of calculating EVPI in Supplementary Fig. 1). We also calculate the relative EVPI for varied management costs and managers’ risk preferences. Our analyses show that learning the feedback information from ecosystem function to species does not improve management outcomes for biodiversity objective, but could improve management outcomes for ecosystem services by up to 231% for risk-prone managers. To illustrate the value of our work to real-world situations, we also apply our approach to an empirical salt marsh ecosystem in Carpinteria Salt Marsh Reserve, CA, USA from Hechinger et al.^46^ and find consistent results with our theoretical findings.

## Results

### The value of feedback information depends on the management objective

**Fig. 2:**
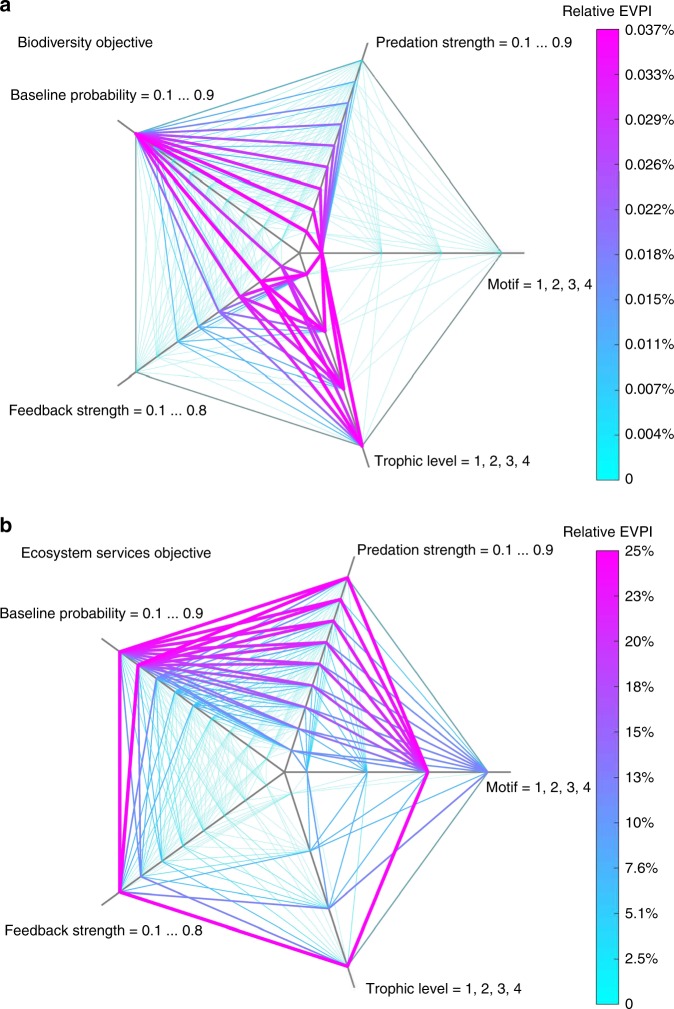
The relative Expected Value of Perfect Information (EVPI) across five ecological features. **a** Ecosystem management for biodiversity objective. **b** Ecosystem management for ecosystem services objective. For each subplot, there are five axis representing five ecological features—motifs, trophic levels, feedback strengths (*α*), species baseline survival probabilities (${\boldsymbol{p}}_{\boldsymbol{j}}^{\mathbf{0}}$), and predation strengths (*b*). The relative EVPI is calculated based on values of those five ecological features (parameters). The parameter values for each axis ranges low to high from the centre to the edge. And the line width is proportional to the value of the relative EVPI

We assess the management benefits of reducing uncertainty for network motifs with different ecological features (see Table 1) under our two different objectives: maximising ecosystem services and species richness. We found that the value of obtaining full knowledge of the feedback links from ecosystem function to species depends on the management objective. Under the management objective of maximising species richness, the relative EVPI is close to zero for all ecological features tested (Fig. 2a). This result suggests that learning this feedback information does not improve the ability to maximise the number of species extant.

**Table 1:** The five ecological features defining an ecosystem configuration in our model

Ecological features	Value tested	Explanation
Motif	1,2,3,4	The network motif of species interactions (1 = linear, 2 = apparent competition, 3 = omnivory, 4 = intraguild competition) (see Fig. 1).
Trophic level	1,2,3,4	The trophic level of the species providing an ecosystem function. The value 1 to 4 represents bottom to top trophic levels (see Fig. 1).
Feedback strength (*α*)	0.1–0.8 by 0.1	The feedback strength represents how much ecosystem function goes back to support species’ survival.
Baseline survival probability $\left( {p_j^0} \right)$	0.1–0.9 by 0.1	The baseline survival probability for species *j* at the initial time step (see Methods).
Predation strength (*b*)	0.1–0.9 by 0.1	The predation strength between two species in the motif network.

We simulated all possible combinations of the features’ values, leading to 10,368 theoretical ecosystems. These ecosystems were used to calculate the Expected Value of Perfect Information (EVPI) under a biodiversity objective and an ecosystem services objective (see Methods)

Under the management objective of maximising ecosystem services, the relative EVPI varies substantially, ranging from 0 to 25% (Fig. 2b). When species’ baseline probabilities of survival ($p_j^0$), feedback strength (*α*), and predation strength (*b*) are small, there is no benefit from knowing the feedback information: applying an optimal strategy with and without knowing the feedback information provides similar ecosystem services outcomes. In contrast, for ecosystem networks with high baseline survival probabilities $\left( {p_j^0} \right)$, high feedback strengths(*α*), and high predation strength (*b*), the relative EVPI could reach up to 25% of improvement of total ecosystem service values (Fig. 2).

### Ecological features of the ecosystem influence the value of feedback information

We study how five ecological features influence the value of knowing the information about feedbacks: motifs, the trophic levels of the species providing the ecosystem function, the proportion of ecosystem function flowing back towards biodiversity (i.e., feedback strength, *α*), the species baseline probability of survival without management ($p_j^0$)^47,48^, and the predation strength between prey and predator(*b*) (see Table 1 and Methods).

**Fig. 3:**
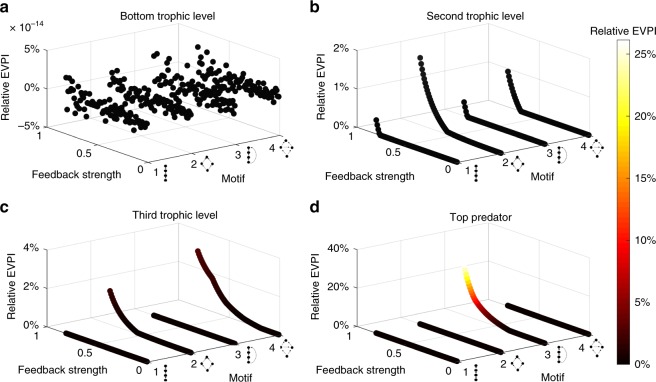
The relative EVPI across the trophic levels of the ecosystem function. Each trophic level is presented in a subplot: **a**–**d** represent trophic levels from low to high. Under ecosystem service objective, we fixed baseline probability of survival to ${\boldsymbol{p}}_{\boldsymbol{j}}^0$ = 0.9, predation strength to *b* = 0.9 and calculated the relative EVPI for all different motifs, trophic levels of the species providing the ecosystem function, and the feedback strength *α*

Among the five ecological features studied, the trophic level of species providing the ecosystem function influenced the value of relative EVPI the most, followed by the species baseline probability of survival ($p_j^0$), network motif, the predation strength (*b*), and the feedback strength (*α*) (see Supplementary Table 1 equal cost conclusion). Although the highest relative EVPI occurs for the omnivory motif (trophic level = 4, $p_j^0$ = 0.8, *α* = 0.8, *b* = 0.9), managers must also consider the influence of other ecological features. For example, the relative EVPI drops from 25% to 0.02% of improvement of ecosystem service values when comparing the top predator providing the ecosystem function (trophic level = 4, EVPI = 25%) versus the secondary consumer providing the ecosystem function (trophic level = 3, EVPI = 0.02%), holding the other parameters constant (Figs. 2b and 3c, d).

The decision tree analysis provides additional insights into the influence of the ecological features on the relative EVPI (Supplementary Fig. 2a). Generally, high relative EVPI (>6%) occurs for omnivory or intraguild competition motifs where higher trophic level species generate the ecosystem function (Supplementary Fig. 2a). In contrast, there is little value to investigate the feedback information when the ecosystem function originates from species at the bottom trophic level, because informed and uninformed strategies have similar management outcomes (Fig. 3a).

### Management cost has little influence on the value of feedback information

We also investigated the influence of management costs on our results. We found that when assuming that higher trophic level species are more expensive to manage, our general conclusions were mostly consistent, with slightly different ordering of which ecological features ranked as important (Supplementary Table 1; Supplementary Figs. 2–4). The trophic level of the species providing the ecosystem function remained the most influential ecological feature for EVPI, while the feedback strength (*α*) became the second important feature, instead of the feature with the lowest EVPI (Supplementary Table 1). The decision tree visualization further shows that, with increased management cost, the highest relative EVPI only occurs when top predator performs the ecosystem function with a high feedback strength (*α* > 0.75) and high baseline survival probability ($p_j^0$ > 0.65) (Supplementary Fig. 2). Overall, we also found that higher management costs of higher trophic level species resulted in higher relative EVPI values for ecosystem services objective (maximum relative EVPI = 72%, Supplementary Fig. 3).

### Salt marsh case study

In the salt marsh ecosystem, we identify four motifs (linear, apparent competition, omnivory, and intraquild competition) between functional groups from Hechinger et al.^46^ with major ecosystem functions (shoreline stabilization, water filtration, and biomass production for fisheries) provided from different trophic levels (mainly bottom and top trophic levels)^34^. For each, we calculate the relative EVPI (see Supplementary Fig. 1 for main steps). We then compare the findings from the empirical case study with our theoretical results to assess if and when feedback information alters optimal management strategies for biodiversity and ecosystem services.

**Fig. 4:**
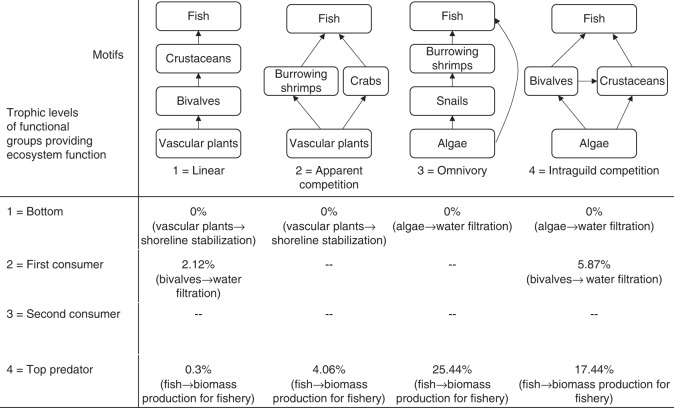
The relative Expected Value of Perfect Information (EVPI) for the salt marsh case study. Four motifs with corresponding ecosystem functions are identified from salt marsh ecosystem in California based on Xiao et al.^34^ and Hechinger et al.^46^. Motifs are listed in columns, and the trophic levels of the functional groups providing ecosystem function are listed in each row. Values indicate the maximum relative EVPI for a specific motif in the column and a specific trophic level in the row. The provisional link from the functional group to ecosystem function is given in brackets under each EVPI value

Results from the salt marsh case study are consistent with our theoretical findings. First, when management objective is maximising biodiversity, the relative EVPIs for all motifs and trophic levels are close to zero. Second, for all four motifs identified from the salt marsh ecosystem, when the ecosystem function is provided by the bottom trophic level (i.e., vascular plants stabilizing the shoreline), the relative EVPIs are close to zero compare to ecosystem function provided by higher trophic levels (bivalves, or fish functional groups) (Fig. 4).

### Risk preferences of managers influence the value of feedback information

The EVPI is an expected value and therefore reflects a risk-neutral decision-maker (indifferent to risk when making decisions)^49^. Decision-makers can also exhibit risk-averse (avoid risk) or risk-prone (seek risk for a higher payoff) preferences and as such we also use the minimax regret criterion^50^, which represents the maximum outcome improvement that could be reached if the feedback information is available (‘Minimal expected regret and min-max regret approaches’ in the Supplementary Methods)^51^. For the biodiversity objective, the maximum regret remains small (from 0.04% to 0.34%, Supplementary Fig. 5a). For the ecosystem service objective, collecting more data could lead to, at best, a maximum regret of 231% ecosystem service improvement compared to no data is collected prior to deciding (Supplementary Fig. 5b). This large improvement in management outcomes occurs in the omnivory motif when the ecosystem function is provided by the top predator (Supplementary Fig. 6a). In this case, uninformed strategies prioritise protecting the basal species while the informed strategies prioritise protecting higher trophic level species (Supplementary Fig. 6c). This difference occurs because one assumes equal probabilities of every possible feedback structures when no information about the true ecosystem function-species feedback structure is available. Therefore, in absence of additional information, protecting the basal species is optimal, to support higher trophic levels for functions and services (Supplementary Fig. 6).

Although we observed that ecological features that have high EVPI have high values of the maximum regret (Fig. 2b, d), these two values do not peak for the same ecological features. For instance, the ecosystem configuration with the highest relative EVPI (omnivory motif, top predator providing the ecosystem function, 80% of function going back to species, 0.8 baseline probability of survival, and 0.9 predation strength, EVPI = 25%, maximum regret = 170%) was not the ecosystem configuration with the highest maximum regret (omnivory motif, with a top predator providing the ecosystem function, 80% of function going back to species, 0.9 baseline probability of survival, and 0.9 predation strength, maximum regret = 231%, EVPI = 24%). Together, the EVPI and maximum regret information provide decision-makers with a richer understanding of the value of reducing uncertainty under different ecosystem structures.

## Discussion

Ecosystem functions not only underpin ecosystem service provision but also provide critical support for species survival. We provide a study investigating the value of knowing part of the ecosystem network structure—the feedbacks from ecosystem function to species—for improving biodiversity or ecosystem services management outcomes. Collecting feedback information is challenging and time consuming, so it is important to find out whether and how much management outcomes could be improved when feedback information is available.

Our results show that knowing the feedback information results in little improvement in biodiversity outcomes yet potentially large improvements for ecosystem services (up to a 25%, Fig. 2). For ecosystem management targeting biodiversity conservation, strategies under perfect information and no information about feedbacks tend to protect the same species: information does not improve the management strategy (Supplementary Fig. 7). In contrast, for management targeting ecosystem services, strategies can be improved by reducing the uncertainty about the ecosystem function-species feedback structure, yet the extent of improvement greatly depends on the particular ecological features (Fig. 3).

Among the five ecological features investigated, the trophic level of the species providing the ecosystem function had the largest impact on EVPI, followed by the baseline probability of survival, the motif structure, the predation strength between species and finally the proportion of ecosystem function going back to biodiversity (Supplementary Table 1). This result has direct implication for managers: by identifying that basal species provides the ecosystem function, decision makers could forgo disentangling complex ecosystem function-species feedbacks for the purpose of improving management decisions, as knowledge of these feedbacks will not improve management outcomes (Fig. 3). The salt marsh case study further supports this result—when basal species (i.e., vascular plants or algae) provide ecosystem functions, such as shoreline stabilization or water filtration, understanding which species or functional groups benefit from the ecosystem function will have little influence on optimal management strategies and outcomes for sequestrated carbon or clean water (Fig. 4). By showing that knowledge of the trophic level of the species providing the ecosystem function improve management outcomes substantially, our study complements the existing literature that have shown that species trophic levels are an important factor for food web stability^52–55^ and for potential trade-offs when managing for biodiversity and ecosystem services^34^.

For management targeting an ecosystem service objective, we identified the ecosystem configuration with the highest relative EVPI (Fig. 2). For the omnivory motif where the top predator performs the ecosystem function, having information about the feedback links from the ecosystem function to species could improve management outcomes by up to 25%—higher than any other motif tested (Figs 2 and 3). These results are consistent with the salt marsh case study when algae, snails, burrowing shrimps, and fish form the omnivory motif with fish providing the ecosystem function (Fig. 4). In this case, the feedbacks could be indirect positive effects of fish biomass on lower trophic levels^56^, or no feedbacks at all in the ecosystem network (subplot (**a**) in Supplementary Fig. 8 and Supplementary Discussion). These results, from our theoretical framework and the case study, prompt important questions for future work, including: how common are these ecosystem structures in nature, and what services are most likely to be produced by such a network structure?

A decision-maker’s risk preferences can influence species protection priorities in conservation^57–59^. Here, we consider both risk preferences and structural uncertainty over ecosystem network to analyse the value of learning the information on feedbacks between ecosystem function and species. We observe that ecosystems with high relative EVPI do not necessarily show high maximum regret for management improvement. A risk-neutral manager would choose to investigate the feedback structures for the ecosystem with the highest expected value, while a risk-prone manager would prefer to learn in another ecosystem with the highest maximum regret.

To gain general mechanistic insights, we considered the stylised system where (1) the network was small (four-nodes motifs) with one ecosystem function and one service provided, (2) learning information about feedbacks had no cost, (3) only trophic interactions were known, and (4) following species losses, no rewiring of the interaction networks was possible. Future work could relax these assumptions. For example, our approach could account for other types of interactions (e.g., parasitism or symbiosis) and larger ecosystem networks with multiple interactions between species and ecosystem functions^26^. Incorporating multiple ecosystem functions will require careful consideration of the management objective due to the increased complexity of interactions between species and functions—should one focus on maximising one particular ecosystem service or ecosystem service bundles^60^? In our optimisation model, we assumed each species contributes to the biodiversity reward function equally, however, managers may assign higher biodiversity reward for protecting specific species (iconic, umbrella, or keystone species), which might lead to a higher EVPI for a biodiversity objective. In contrast, for the ecosystem services objective, we assumed no substitutability between species in the delivery of services. However, in many terrestrial ecosystems, ecosystem functions show resilience because several species can perform the same ecosystem function^61^. In this case, a lower EVPI might be expected for ecosystem services objective. In particular, for an ecosystem where all components of the system provide the same ecosystem function (e.g., multiple plant species can provide habitat for birds and pollinators), future work could investigate the value of information for not only which species benefit from the ecosystem function (the location of the feedback links) but also on the relative importance of those links because of the potential for interspecies competition, complementarity and substitutability^61^.

We assumed equal management cost for each species; however, management cost for species in higher trophic levels of the food web could be higher, because these species could experience higher extinction rates, requiring more costly interventions to maintain their populations (^62^ but see^63^ for a counter example), or require protection of more area due to their larger ranges^64^. For completion, we also analysed the influence of increased management cost for species in higher trophic levels and found similar results (Supplementary Table 1). Practically, ecosystem manager would also have to consider whether feedback information from ecosystem function to species could be easily collected−in other words, is this uncertainty reducible, and is reducing it cost-effective? Information about feedbacks between ecosystem functions and species could be difficult to detect from the ecosystem dynamics and the field data. Quantifying if and how much a species benefits from or depends on particular functions of the system is even more challenging^65,66^. For biodiversity objective, managers would not choose to reduce the feedback uncertainty no matter of the cost of collecting the information, because there is little biodiversity outcome improvement with feedback information (relative EVPI close to zero). However, for ecosystem services objective, managers may need to explicitly consider the ecological features of the ecosystem and balance the costs of acquiring information with the management returns from having that information. Further research on case studies which examine the cost of monitoring and field work would complement our recommendations^67^.

Both the production of ecosystem services and the protection of nature are key aims for ecosystem management. The tangled web of connections and feedbacks within ecosystems clearly complicate management decisions for both aims. Knowing when additional information about these connections is warranted helps inform more effective decisions that can protect species and the services on which society relies. Our study provides an approach, combining network theory and optimisation techniques, to assess the importance of learning about the connections in ecosystems with feedbacks between ecosystem function and species prior to making costly decisions. By quantifying the value of information about feedbacks information, in terms of improved outcomes for biodiversity conservation and ecosystem services for numerous ecosystem configurations, we provide a scaffold for scientists and managers to discern the circumstances in which learning this information would be most promising, and thus help take a further step towards better ecosystem management for species and ecosystem services around the world.

## Methods

### Overview

To determine whether collecting data on feedback information results in management improvement, we use network motifs to capture the species interactions and we model the ecosystem dynamics on a network motif, the ecosystem function and the services. While biodiversity is a service in its own right^68^, we consider other types of services (e.g. provisioning, regulating and supporting services) which depend on the structure and processes of species and their interactions^69^. We assume that without management, each species has a probability of extinction at each time step and managers must decide which species to manage. We do not know which species benefit from the ecosystem function (Supplementary Fig. 8), and we calculate the Expected Value of Perfect Information (EVPI) to determine the value of resolving this uncertainty. Then, we investigate how EVPI changes across five ecological features: different motifs, trophic level of the function, feedback strengths (*α*), baseline probabilities of survival ($p_j^0$), and predation strength(*b*). In addition, we compare the ecological features of the maximum EVPI, also called the ‘expected regret’^50^, with those features of the ‘maximum regret’ that a manager could have when the feedback information is not available (‘Minimal expected regret and min-max regret approaches’ in the Supplementary Methods).

### Network motifs

We consider network motifs with four nodes connected to one ecosystem function and one service (Fig. 1). A node represents a species, and its position in the motif represents the species trophic level from low (basal species) to high (top predator). We assume that species’ feeding relationships and the provisional links from species to ecosystem function and services are known, but we have uncertainty over the different possible combinations of species that benefit from the ecosystem function. In other words, the ecosystem function could benefit either one, two, three, or all species in the motif, resulting in 16 possible structures per motif (Supplementary Fig. 8). We assume that, for a fixed amount of a function provided by species, there is a trade-off between its provision for services and feedback for biodiversity. For example, the more freshwater is taken out of a stream for irrigation purpose, the less water is left to support biodiversity in that ecosystem^70^. We also assume that for the amount of ecosystem function going back, species will consume these feedbacks equally.

The dynamics and management problem of our ecosystem networks are modelled as Markov Decision Processes (MDPs, see below subsection ‘Ecosystem dynamics and transition probabilities’ and ‘Using Markov Decision Processes to model species dynamics and protection actions effects’ in the Supplementary Methods). Building on previous work by Xiao et al.^34^, we have added the interactions between species and ecosystem functions, and top-down effects (i.e. the probability of survival of a species depends on predators and preys neighbourhoods, see ‘Using Markov Decision Processes to model species dynamics and protection actions effects’ in the Supplementary Methods).

### Management actions

We assume that managers can protect one species at each time step, with the same management cost for each species (we run further analysis with increased management cost as the trophic level of the species increases, Supplementary Table 1). We define a strategy *δ* as a function that prescribes which species to protect for a given ecosystem state (defined by the set of species extant in the ecosystem). The strategy is applied in the initial ecosystem state, at the next time-step some species become extinct while other remain extant. The strategy then is applied to this new ecosystem state, and so on. The sequential application of the strategy defines a sequence of species to protect.

An optimal strategy is defined as a strategy that yields the maximum level of the expected outcome (see ‘Value of a strategy, δ’ in the Supplementary Methods). Here, we consider two definitions of the outcome: the discounted sum of expected number of extant species across all possible states in the system, and the discounted sum of expected amount of ecosystem service provided by the system (measured in US dollars).

### Ecosystem dynamics and transition probabilities

The ecosystem dynamics are captured in the transition probability matrix in MDP. Let P be the transition probability matrix representing the dynamics of the system from time step *t* to time step *t* + 1. $P\left( {x^{t + 1}{\mathrm{|}}x^t,a^t,{\mathrm{p}}_{\mathrm{j}}^0,\alpha ,b,f,M} \right)$ represents the conditional probability of the ecosystem transitioning from state *x*^*t*^ to *x*^*t*+1^ given action *a*^*t*^ is implemented at time *t*. We assume that species *j* could be present ($x_j^t = 1$) or absent ($x_j^t = 0$) at each time step. This transition probability is also conditional on the baseline probability of survival of species *j* ${\mathrm{p}}_{\mathrm{j}}^0$, the feedback strength *α* (the percentage of the ecosystem function going back to a species), the predation strength *b*, the feedback structure*f* and the food web matrix M representing the prey-predator interactions of our system. To model this transition probability, we assume that, knowing the state at time *t*, *x*^*t*^, the state of species *j* at time *t* + 1 is independent of the state of the other species at time *t* + 1. So we can define the transition probability *P*as the product of*J*individual species’ transition probabilities:$$\begin{array}{l}P\left( {x^{t + 1}{\mathrm{|}}x^t,a^t,{\mathrm{p}}_{\mathrm{j}}^0,\alpha ,b,f,M} \right) = \\ \mathop {\prod}\nolimits_{j = 1}^J {P_j\left( {x_j^{t + 1}|x^t,a^t,{\mathrm{p}}_{\mathrm{j}}^0,\alpha ,b,f,M} \right)} \end{array}$$.

Survival probability of a species will increase with the number of extant preys*N*_*prey*_(*j*,*x*^*t*^,*M*) and ecosystem function available *N*_*EF*_ (*j*,*x*^*t*^,*f*), and will decrease with the number of extant predators *N*_*predator*_(*j*,*x*^*t*^,*M*). We assume that *N*_*prey*_(*j*, x^t^, *M*), *N*_*EF*_(*j*, x^t^, *f*), and *N*_*predator*_(*j*, *x*^*t*^, *M*) are maximum at the initial time step where all species are present (i.e. *x*^*t*^ = *x*^0^ = [1,1,1,1]). Formally, we defined the transition probability when species *j* is not under protection (*a*^*t*^ ≠ *j*) as the product of four terms:

1 $$\begin{array}{l}P_j^t\left( {x_j^{t + 1} = 1{\mathrm{|}}x_j^t = 1,a^t \, \ne \, j,{\mathrm{p}}_{\mathrm{j}}^0,\alpha ,b,f,M} \right) \\ = p_j^0 \ast \frac{{N_{prey}\left( {j,x^t,M} \right)}}{{N_{prey}\left( {j,x^0,M} \right)}} \ast \left( {1 - b\frac{{N_{predator}\left( {j,x^t,M} \right)}}{{N_{predator}\left( {j,x^0,M} \right)}}} \right)\\ \ast \frac{{N_{EF}\left( {j,x^t,f,\alpha } \right)}}{{N_{EF}^ \ast \left( {j,f} \right)}}\end{array}$$

In this way, under the most favourable condition where species *j* has no predator, no prey loss and receive maximum level of ecosystem function, the above equation reduces to its baseline probability of survival $p_j^0$. However, species *j* survival probability will decrease when at least one of the following three events happen—prey loss, predator presence, or insufficient functional support (see ‘Using Markov Decision Processes to model species dynamics and protection actions effects’ in the Supplementary Methods).

### Calculating the Expected Value of Perfect Information (EVPI)

The value of Information can be determined by calculating the Expected Value of Perfect Information (EVPI)^35,71,72^. The EVPI is an indicator of how much additional value, in expectation, can be gained by knowing ecosystem function-species feedback^44^. Formally, EVPI is the expected difference between the outcome of the optimal ‘informed’ strategy when we have full feedback information (*EV*_*certainty*_) and the outcome of the optimal ‘uninformed’ strategy when we have no feedback information from the ecosystem function to species (*EV*_*uncertainty*_). Here, *EV* is in terms of the number of species preserved or the ecosystem service values as mentioned above,

2 $$EVPI = EV_{certainty} - EV_{uncertainty}$$

3 $${\mathrm{with}}\,EV_{certainty} = E_f\left[ {{\mathrm{max}}_{{\mathrm{\delta }}_{\mathrm{f}}}{\mathrm{V}}_{\delta _f}\left( {x^0,f} \right)} \right] = E_f\left[ {{\mathrm{V}}_{\delta _f^ \ast }\left( {x^0,f} \right)} \right]$$

*EV*_*certainty*_ represents the expected value of an optimal informed strategy when we have full information (Supplementary Fig. 6a). Here, $\delta _f^ \ast$ is a strategy that maximises *V*_*δ*_(*x*^0^,*f*), the value when the initial ecosystem state is *x*^0^ and the feedback structure is *f* (see ‘Using Markov Decision Processes to model species dynamics and protection actions effects’ in the Supplementary Methods). Therefore, *EV*_*certainty*_ corresponds to the situation where the manager will first discover which structure is true, then will implement the best management strategy for this structure. To compute it, first, we calculate the optimal strategy $\delta _f^ \ast$ for each possible feedback structure*f* (See next paragraph). Second, we calculate the expectation of the optimal value obtained when applying the optimal strategy, across the possible feedback structures. Without a priori information, we assume a uniform prior over the feedback structures: $E_f\left[ {V_{\delta _f^ \ast }\left( {x^0,f} \right)} \right] = \frac{1}{F}\mathop {\sum}\nolimits_{f = 1}^F {V_{\delta _f^ \ast }\left( {x^0,f} \right)}$.

This calculation requires solving F optimisation problems to find the F optimal informed strategies $\delta _f^ \ast$. Each optimisation problem is a classical problem of dynamic programming optimisation^73^, see full details of MDP models in ‘Using Markov Decision Processes to model species dynamics and protection actions effects’ in the Supplementary Methods). We solve each MDP using a *Policy Iteration* approach, implemented in the MDPtoolbox^74^.

Under uncertainty, the manager must choose a management strategy without knowing which feedback structure applies to its ecosystem (Supplementary Fig. 6b). We assume the manager chooses the one that maximises the expected value of a strategy across all possible feedback structures, which is defined as:

4 $$EV_{uncertainty} = \max _\delta E_f\left[ {V_\delta \left( {x^0,f} \right)} \right]\\ = \mathop {{{\mathrm{max}}}}\limits_{\mathrm{\delta }} \frac{1}{F}\mathop {\sum}\nolimits_{f = 1}^F {V_\delta \left( {x^0,f} \right)} = \frac{1}{F}\mathop {\sum}\nolimits_f {V_{\delta ^ \ast }\left( {x^0,f} \right)}$$

where δ^*^ is a strategy that achieves the maximum expected value under uncertainty, and is called the optimal uninformed strategy.

Finding the optimal strategy under uncertainty *EV*_*uncertainty*_ by solving Equation (4) is a hard-combinatorial problem. There is now a single optimisation problem but it does not correspond anymore to solving multiple MDP problems. Policy Iteration or more generally dynamic programming approaches, which can be used for solving MDPs, cannot be used for solving Equation (4), where the feedback structure is unknown. As a result, unlike when the feedback structure is known, the term $\frac{1}{F}\mathop {\sum }\limits_{f = 1}^F V_\delta \left( {x^0,f} \right)$ has to be evaluated for all candidate strategies in order to determine the optimal one. In our case, computing *EV*_*uncertainty*_ requires evaluating 4^16^ strategies under 16 different ecosystem structures, which is computationally time consuming. For this reason, instead of evaluating each possible strategy, we only evaluate the 16 optimal strategies $\delta _f^ \ast$ derived from solving the MDP for each of the 16 possible structures in the certainty case. We then choose the best amongst these. In doing so, we assume the optimal strategy under uncertainty is one of the 16 optimal strategies $\delta _f^ \ast$ derived under certainty, which is an approximation. For completeness, we also compare our approximation with a genetic algorithm solution (see ‘Genetic Algorithm approximation’ in the Supplementary Methods). No major differences were found, which indicates that MDP strategies perform as well as solutions of a genetic algorithm in this case (Supplementary Fig. 9).

### Influence of ecological features on EVPI

To evaluate the influence of ecological features on the value of information for management outcomes, we calculate and analyse the EVPI for different values of five features (see Table 1): motif type, trophic level, feedback strength (α), baseline probability of survival ($p_j^0$), and predation strength (*b*), for both biodiversity and ecosystem service objectives. When combining all possible values of these features, we obtain 10,368 different ecosystem configurations. We calculate the EVPI and the maximum regret for each objective and ecosystem configuration. To understand how different ecological features affect the EVPI, we perform a decision tree analysis (Supplementary Table 1, Supplementary Fig. 1). To evaluate how the feedback strength (α) affects the EVPI, under an ecosystem service objective, we fix $p_j^0$ = 0.9, *b* = 0.9, and vary α between 0.1 to 0.8 by 0.01 intervals (Fig. 3).

### Salt marsh case study

We apply the same approach as in the simulations on an empirical salt marsh ecosystem that provides ecosystem functions and services, based on Xiao et al.^34^ and Hechinger et al.^46^. The salt marsh food web consists of 12 functional groups with four types of ecosystem functions^34^, including carbon sequestration (i.e., provided by vascular plants), water filtration (e.g., provided by bivalves), shoreline stabilization (e.g., provided by vascular plants), and biomass production for fisheries (i.e., provided by upper trophic levels) (Supplementary Fig. 10). From that food web, we identified four motifs—linear, apparent competition, omnivory, and intraguild competition with corresponding ecosystem functions (Fig. 4, ‘Salt marsh case study’ in the Supplementary Methods).

We use the approach from Xiao et al.^34^ to calculate the baseline survival probabilities for each functional group (represented as a node in the motif). As there is no empirical data on the feedback strength and predation strength, we simulate a wide range of values for the feedback strength α (between 0.1 and 0.8 by 0.01 intervals) and predation strength *b* (between 0.1 and 0.9 by 0.01 intervals). We calculate the relative EVPI for each motif and each trophic level if that trophic level is associated with ecosystem function provision (see Supplementary Fig. 1 for details). We then calculate the maximum value across different values in $p_j^0$, *a* and *b* for each motif and trophic levels from this salt marsh network.

### Reporting Summary

Further information on research design is available in the Nature Research Reporting Summary linked to this article.

## Supplementary information

Supplementary Information

Reporting Summary

## Data Availability

All data to support the conclusions in this paper are available in the main text or the supplementary materials.

## Code availability

All simulations were conducted in Matlab and R. The source code to reproduce our results for equal management cost is available at 10.6084/m9.figshare.7712090.v1^75^. And the source code to reproduce our results for increased management cost is available at 10.6084/m9.figshare.6668087.v1^76^.

## References

[1] Tallis H, Kareiva P, Marvier M, Chang A (2008). An ecosystem services framework to support both practical conservation and economic development. Proc. Natl Acad. Sci. USA.

[2] Turner WR (2007). Global conservation of biodiversity and ecosystem services. BioScience.

[3] Balvanera P (2014). Linking biodiversity and ecosystem services: current uncertainties and the necessary next steps. BioScience.

[4] Dee LE, De Lara M, Costello C, Gaines SD (2017). To what extent can ecosystem services motivate protecting biodiversity?. Ecol. Lett..

[5] Thompson RM (2012). Food webs: reconciling the structure and function of biodiversity. Trends Ecol. Evol..

[6] Bruno JF, Stachowicz JJ, Bertness MD (2003). Inclusion of facilitation into ecological theory. Trends Ecol. Evol..

[7] Wright, A. J., Wardle, W. D. A., Callaway, W. R. & Gaxiola, A. The overlooked role of facilitation in biodiversity experiments. *Trends in Ecol. Evol.* **32**, 383–390 (2017). 10.1016/j.tree.2017.02.011 28283253

[8] Kleijn D (2015). Delivery of crop pollination services is an insufficient argument for wild pollinator conservation. Nat. Commun..

[9] Muscatine L, Porter JW (1977). Reef corals: mutualistic symbioses adapted to nutrient-poor environments. Bioscience.

[10] Polovina JJ (1984). Model of a coral reef ecosystem. Coral reefs.

[11] Henry L-A (2013). Cold-water coral reef habitats benefit recreationally valuable sharks. Biol. Conserv..

[12] Suttle KB (2003). Pollinators as mediators of top‐down effects on plants. Ecol. Lett..

[13] Moore, F. & Best, B. in *Global Trade and Consumer Choices: Coral Reefs in Crisis*, *Proceedings of an American Association for the Advancement of Science (AAAS) Meeting*. 5–10.

[14] Bellwood DR, Hughes TP, Folke C, Nyström M (2004). Confronting the coral reef crisis. Nature.

[15] Cox PA, Elmqvist T (2000). Pollinator extinction in the Pacific Islands. Conserv. Biol..

[16] Cox Paul Alan (1983). Extinction of the Hawaiian Avifauna Resulted in a Change of Pollinators for the ieie, Freycinetia arborea. Oikos.

[17] Macfadyen S (2009). Do differences in food web structure between organic and conventional farms affect the ecosystem service of pest control?. Ecol. Lett..

[18] Truchy A, Angeler DG, Sponseller RA, Johnson RK, McKie BG (2015). Chapter two-linking biodiversity, ecosystem functioning and services, and ecological resilience: towards an integrative framework for improved management. Adv. Ecol. Res..

[19] Pinto R, de Jonge VN, Marques JC (2014). Linking biodiversity indicators, ecosystem functioning, provision of services and human well-being in estuarine systems: application of a conceptual framework. Ecol. Indic..

[20] Loreau M (2001). Biodiversity and ecosystem functioning: current knowledge and future challenges. Science.

[21] Harrison P (2014). Linkages between biodiversity attributes and ecosystem services: a systematic review. Ecosyst. Serv..

[22] Bastolla U (2009). The architecture of mutualistic networks minimizes competition and increases biodiversity. Nature.

[23] Bascompte J, Jordano P, Olesen JM (2006). Asymmetric coevolutionary networks facilitate biodiversity maintenance. Science.

[24] Kéfi S (2015). Network structure beyond food webs: mapping non-trophic and trophic interactions on Chilean rocky shores. Ecology.

[25] Kéfi S (2012). More than a meal… integrating non-feeding interactions into food webs. Ecol. Lett..

[26] Ings TC (2009). Ecological networks–beyond food webs. J. Anim. Ecol..

[27] Barbier EB (2000). Valuing the environment as input: review of applications to mangrove-fishery linkages. Ecol. Econ..

[28] Kellner JB, Sanchirico JN, Hastings A, Mumby PJ (2011). Optimizing for multiple species and multiple values: tradeoffs inherent in ecosystem-based fisheries management. Conserv. Lett..

[29] Dee LE (2017). Operationalizing network theory for ecosystem service assessments. Trends Ecol. Evol..

[30] Pikitch E (2004). Ecosystem-based fishery management. Science.

[31] Reyers B, Polasky S, Tallis H, Mooney HA, Larigauderie A (2012). Finding common ground for biodiversity and ecosystem services. BioScience.

[32] Fisher B (2011). Impacts of species-led conservation on ecosystem services of wetlands: understanding co-benefits and tradeoffs. Biodivers. Conserv..

[33] Gascuel D, Pauly D (2009). EcoTroph: modelling marine ecosystem functioning and impact of fishing. Ecol. Model..

[34] Xiao H (2018). Win-wins for biodiversity and ecosystem service conservation depend on the trophic levels of the species providing services. J. Appl. Ecol..

[35] Schlaifer, R. & Raiffa, H. *Applied statistical decision theory*. (1961).

[36] Marescot L (2013). Complex decisions made simple: a primer on stochastic dynamic programming. Methods Ecol. Evol..

[37] Sabbadin R, Spring D, Rabier C-E (2007). Dynamic reserve site selection under contagion risk of deforestation. Ecol. Model..

[38] Polasky S, Solow AR (2001). The value of information in reserve site selection. Biodivers. Conserv..

[39] Milo R (2002). Network motifs: simple building blocks of complex networks. Science.

[40] Bascompte J (2009). Disentangling the web of life. Science.

[41] Stouffer DB, Bascompte J (2010). Understanding food-web persistence from local to global scales. Ecol. Lett..

[42] Stouffer DB, Camacho J, Jiang W, Amaral LAN (2007). Evidence for the existence of a robust pattern of prey selection in food webs. Proc. R. Soc. Lond. B: Biol. Sci..

[43] Chadès I (2011). General rules for managing and surveying networks of pests, diseases, and endangered species. Proc. Natl Acad. Sci. USA.

[44] Williams BK, Johnson FA (2015). Value of information and natural resources decision-making. Wildl. Soc. Bull..

[45] Canessa S (2015). When do we need more data? A primer on calculating the value of information for applied ecologists. Methods Ecol. Evol..

[46] Hechinger RF (2011). Food webs including parasites, biomass, body sizes, and life stages for three California/Baja California estuaries: Ecological Archives E092-066. Ecology.

[47] Probert, W. J., McDonald-Madden, E., Peyrard, N. & Sabbadin, R. in *Proceedings of the European Conference on Artificial Intelligence*.

[48] Eklöf A, Tang S, Allesina S (2013). Secondary extinctions in food webs: a Bayesian network approach. Methods Ecol. Evol..

[49] Von Neumann, J. & Morgenstern, O. *Theory of Games and Economic Behavior*. (Princeton university press, 2007).

[50] Bell DE (1982). Regret in decision making under uncertainty. Oper. Res..

[51] Gardner M, Steinberg L (2005). Peer influence on risk taking, risk preference, and risky decision making in adolescence and adulthood: an experimental study. Dev. Psychol..

[52] Nichols S, Garling D (2000). Food-web dynamics and trophic-level interactions in a multispecies community of freshwater unionids. Can. J. Zool..

[53] Binzer A (2011). The susceptibility of species to extinctions in model communities. Basic Appl. Ecol..

[54] Binzer A, Guill C, Brose U, Rall BC (2012). The dynamics of food chains under climate change and nutrient enrichment. Philos. Trans. R. Soc. B: Biol. Sci..

[55] Johnson S, Domínguez-García V, Donetti L, Muñoz MA (2014). Trophic coherence determines food-web stability. Proc. Natl. Acad. Sci. USA.

[56] Frank KT, Petrie B, Choi JS, Leggett WC (2005). Trophic cascades in a formerly cod-dominated ecosystem. Science.

[57] Tulloch AI (2015). Effect of risk aversion on prioritizing conservation projects. Conserv. Biol..

[58] Hammill E, Tulloch A, Possingham H, Strange N, Wilson K (2016). Factoring attitudes towards armed conflict risk into selection of protected areas for conservation. Nat. Commun..

[59] Mouysset L, Doyen L, Jiguet F (2013). How does economic risk aversion affect biodiversity?. Ecol. Appl..

[60] Raudsepp-Hearne C, Peterson GD, Bennett E (2010). Ecosystem service bundles for analyzing tradeoffs in diverse landscapes. Proc. Natl Acad. Sci. USA.

[61] Batabyal AA (1999). Species substitutability, resilience, and the optimal management of ecological-economic systems. Math. Comput. Model..

[62] Purvis A, Gittleman JL, Cowlishaw G, Mace GM (2000). Predicting extinction risk in declining species. Proc. R. Soc. Lond. Ser. B: Biol. Sci..

[63] Eklöf A, Ebenman B (2006). Species loss and secondary extinctions in simple and complex model communities. J. Anim. Ecol..

[64] McDonald-Madden, E. *et al*. Using food-web theory to conserve ecosystems. *Nature Communications* **7**, 10245, (2016). 10.1038/ncomms10245 PMC4735605 26776253

[65] Duncan, C., Thompson, J. R. & Pettorelli, N. in *Proc. R. Soc. B*. 20151348 (The Royal Society).

[66] Fisher B, Turner RK (2008). Ecosystem services: classification for valuation. Biol. Conserv..

[67] Nadiminti R, Mukhopadhyay T, Kriebel CH (1996). Risk aversion and the value of information. Decis. Support Syst..

[68] Mace GM, Norris K, Fitter AH (2012). Biodiversity and ecosystem services: a multilayered relationship. Trends Ecol. Evol..

[69] Haines-Young, R. & Potschin, M. The links between biodiversity, ecosystem services and human well-being. *Ecosystem Ecology: a new synthes**is.* **1**, 110–139 (2010).

[70] Dudgeon D (2006). Freshwater biodiversity: importance, threats, status and conservation challenges. Biol. Rev..

[71] Howard RA (1966). Information value theory. IEEE Trans. Syst. Sci. Cybern..

[72] Yokota F, Thompson KM (2004). Value of information analysis in environmental health risk management decisions: past, present, and future. Risk Anal..

[73] Bertsekas DP (1996). Dynamic programming and optimal control. J. Oper. Res. Soc..

[74] Chadès I, Chapron G, Cros MJ, Garcia F, Sabbadin R (2014). MDPtoolbox: a multi‐platform toolbox to solve stochastic dynamic programming problems. Ecography.

[75] Xiao, H. VOI paper equal cost. figshare. Fileset., 10.6084/m9.figshare.7712090.v1 (2019).

[76] Xiao, H. VOI of feedbacks. figshare. Code, 10.6084/m9.figshare.6668087.v1 (2018).

