# Supplementary material for: The value of understanding feedbacks from ecosystem functions to species for managing ecosystems
Source: Nat Commun. 2019 Aug 29;10:3901. doi: 10.1038/s41467-019-11890-7 (PMC6715698; doi:10.1038/s41467-019-11890-7)
Supplement: Supplementary file 1 — Supplementary Information [file 41467_2019_11890_MOESM1_ESM.pdf]

The value of understanding feedbacks from ecosystem functions to species for managing ecosystems

Xiao et al.

Supplementary Information

Supplementary Figures

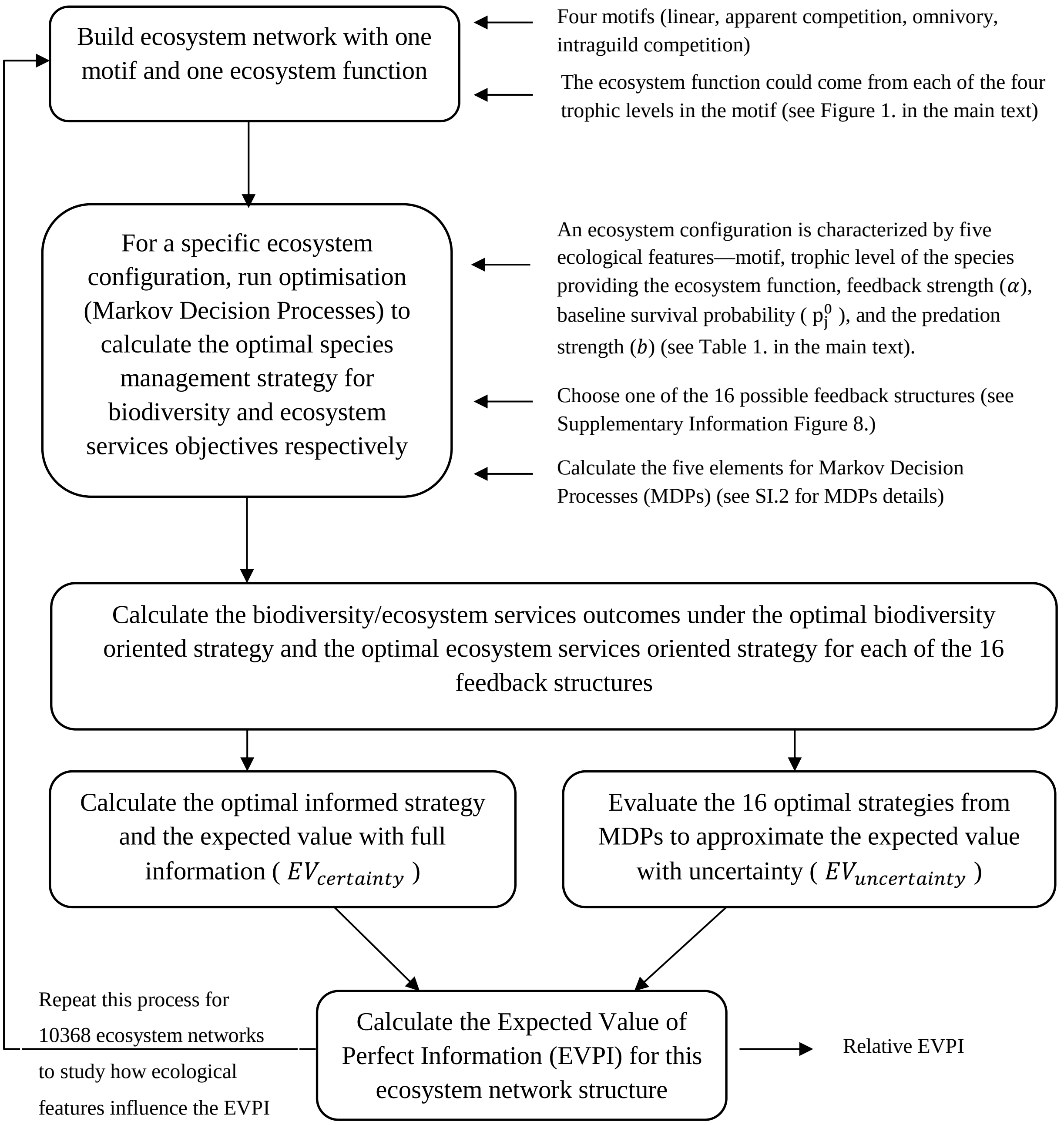

Supplementary Figure 1

Main steps to calculate the Expected Value of Perfect Information (EVPI).

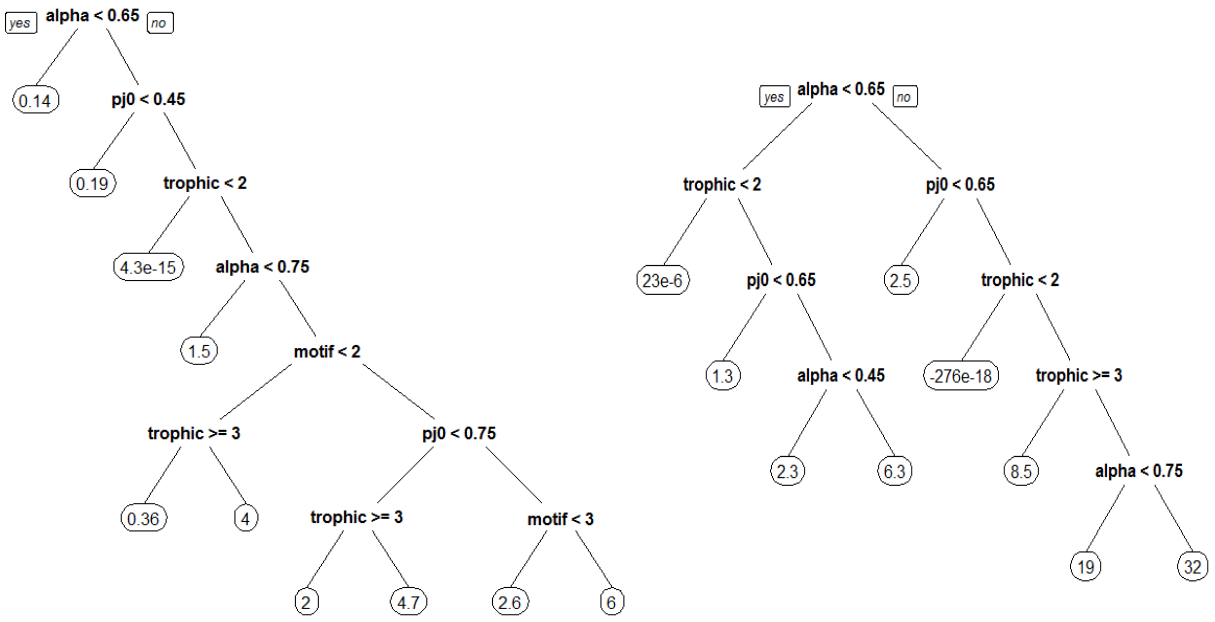

a Equal management cost for each species

b Increased management cost as the trophic level of the species increases

Supplementary Figure 2

Decision tree comparison on the relative EVPI between equal management cost and increased management cost. Under ecosystem services objective, **a** shows the decision tree under equal management cost assumption, and **b** shows the tree under increased management cost assumption with the same parameters as the decision tree analysis in subplot **a**. Leaves in the decision tree are the predicted value of the relative EVPI.

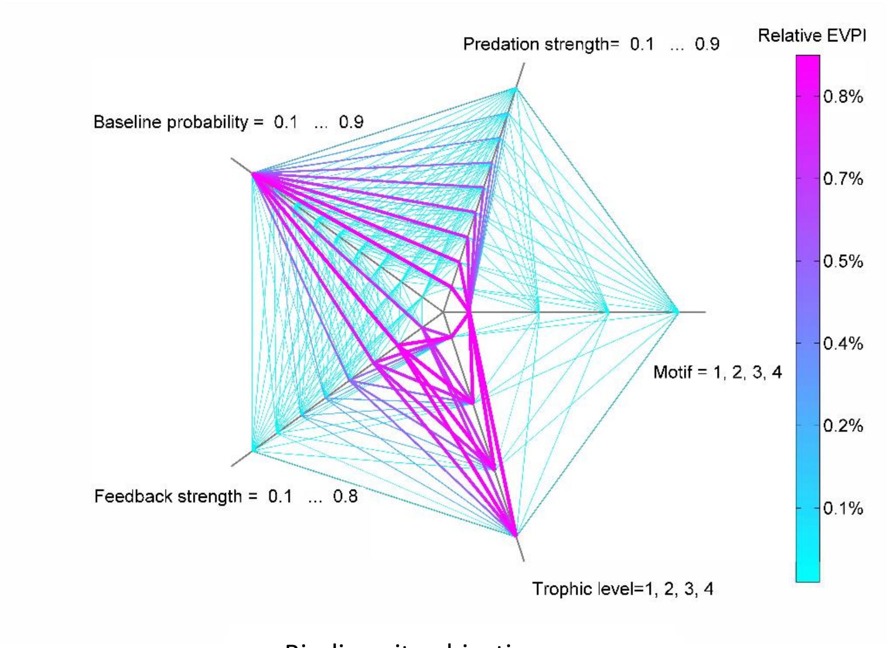

a Biodiversity objective

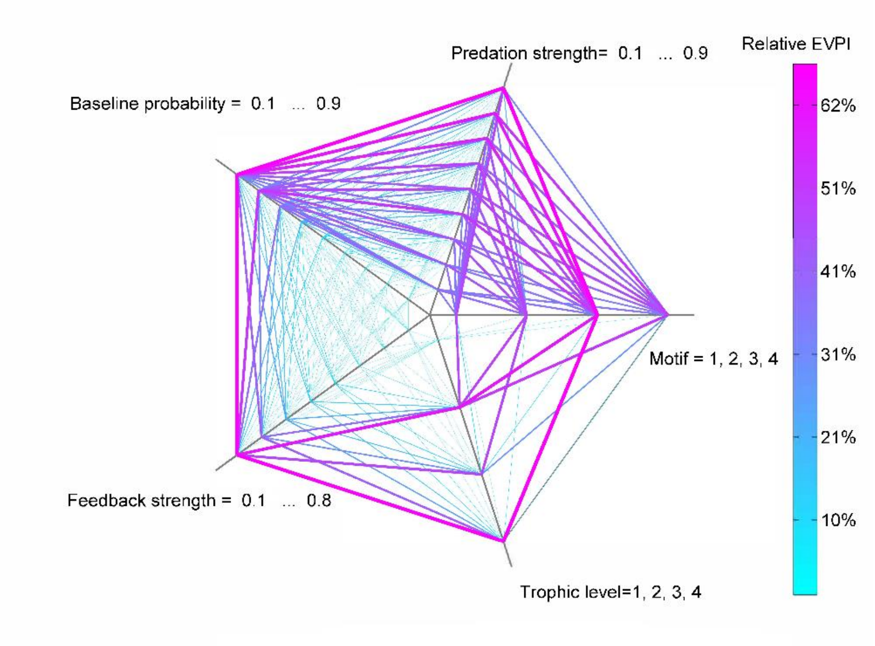

b Ecosystem services objective

Supplementary Figure 3

The relative Expected Value of Perfect Information (EVPI) across five ecological features, with increased management cost as trophic level of the species increases. **a** Ecosystem management for biodiversity objective. **b** Ecosystem management for ecosystem services objective. Each axis dimension represents one of the five parameters (ecological features), with parameter values ranging low to high from the centre to the edge. The line width is proportional to the value of the relative EVPI.

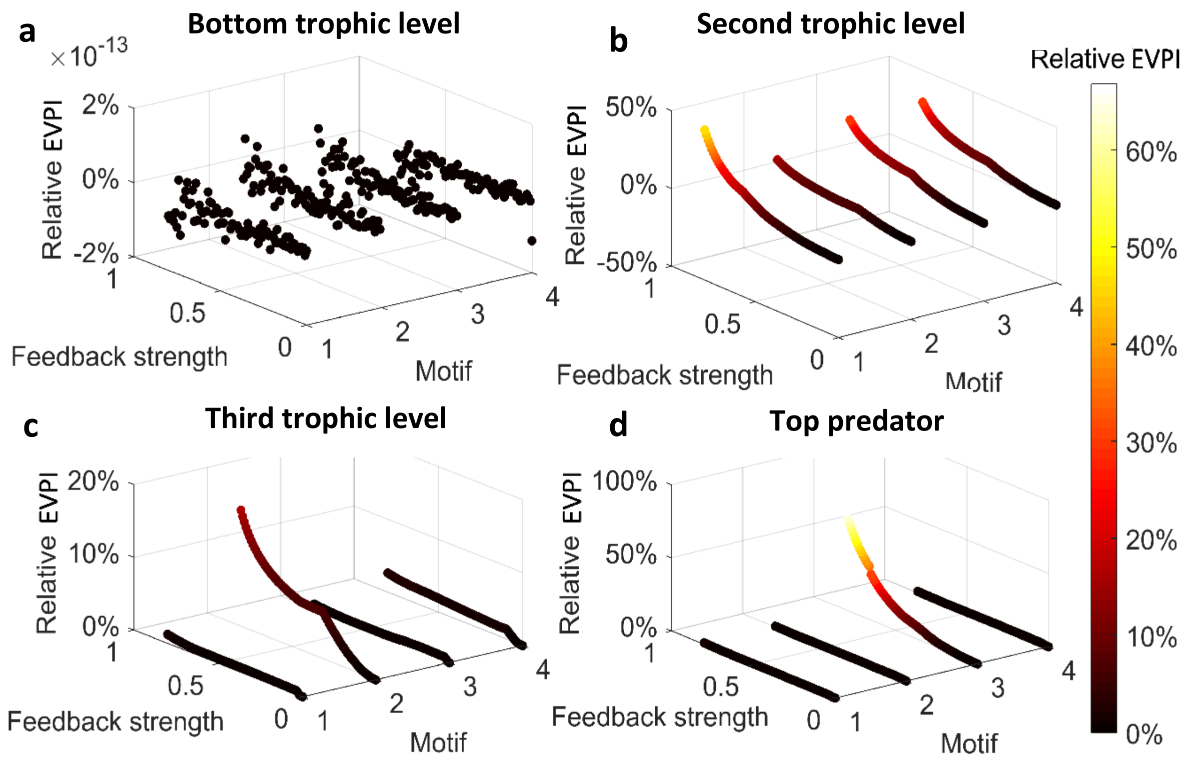

Supplementary Figure 4

The relative EVPI across the trophic levels of the ecosystem function, with increased management cost as trophic level of the species increases. Each trophic level is presented in a subplot: **a-d** represent trophic levels from low to high. Under ecosystem service objective, we fixed baseline probability of survival to $\boldsymbol{p_j^0}$ =0.9, predation strength to b=0.9 and calculate the relative EVPI for all different motifs, trophic levels of the species providing the ecosystem function, and the feedback strength $\boldsymbol{\alpha}$.

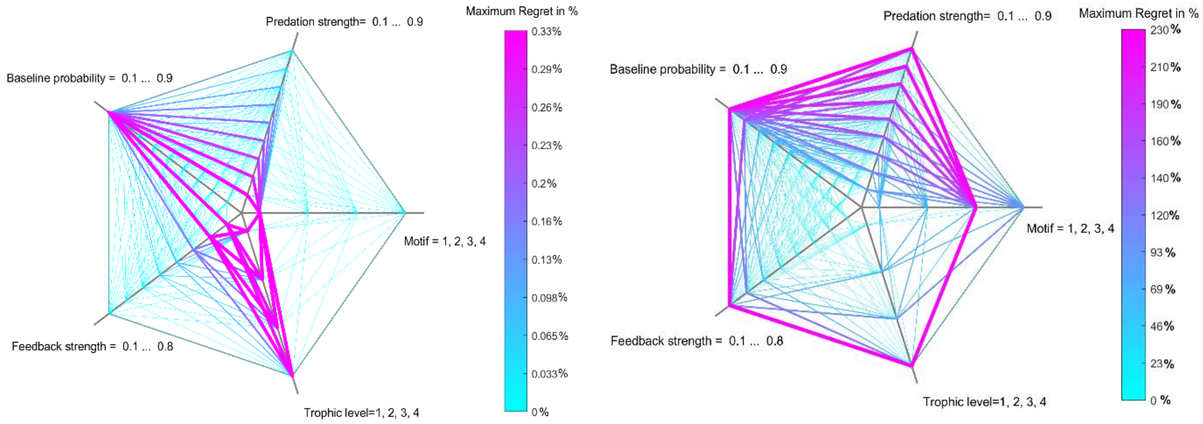

a Biodiversity objective

b Ecosystem services objective

Supplementary Figure 5

The Maximum Regrets under biodiversity and ecosystem services objectives (with equal management cost). Five axes represent five ecological features—motifs, trophic levels of the ecosystem function, feedback strength ($\boldsymbol{\alpha}$), baseline probability of survival ($\boldsymbol{p_j^0}$), and predation strength (b), with values low to high from centre to edge. Lines inside the radar plot represent the Maximum Regret values in percentage, as the colour bar indicated, and the line width is proportional to the value.

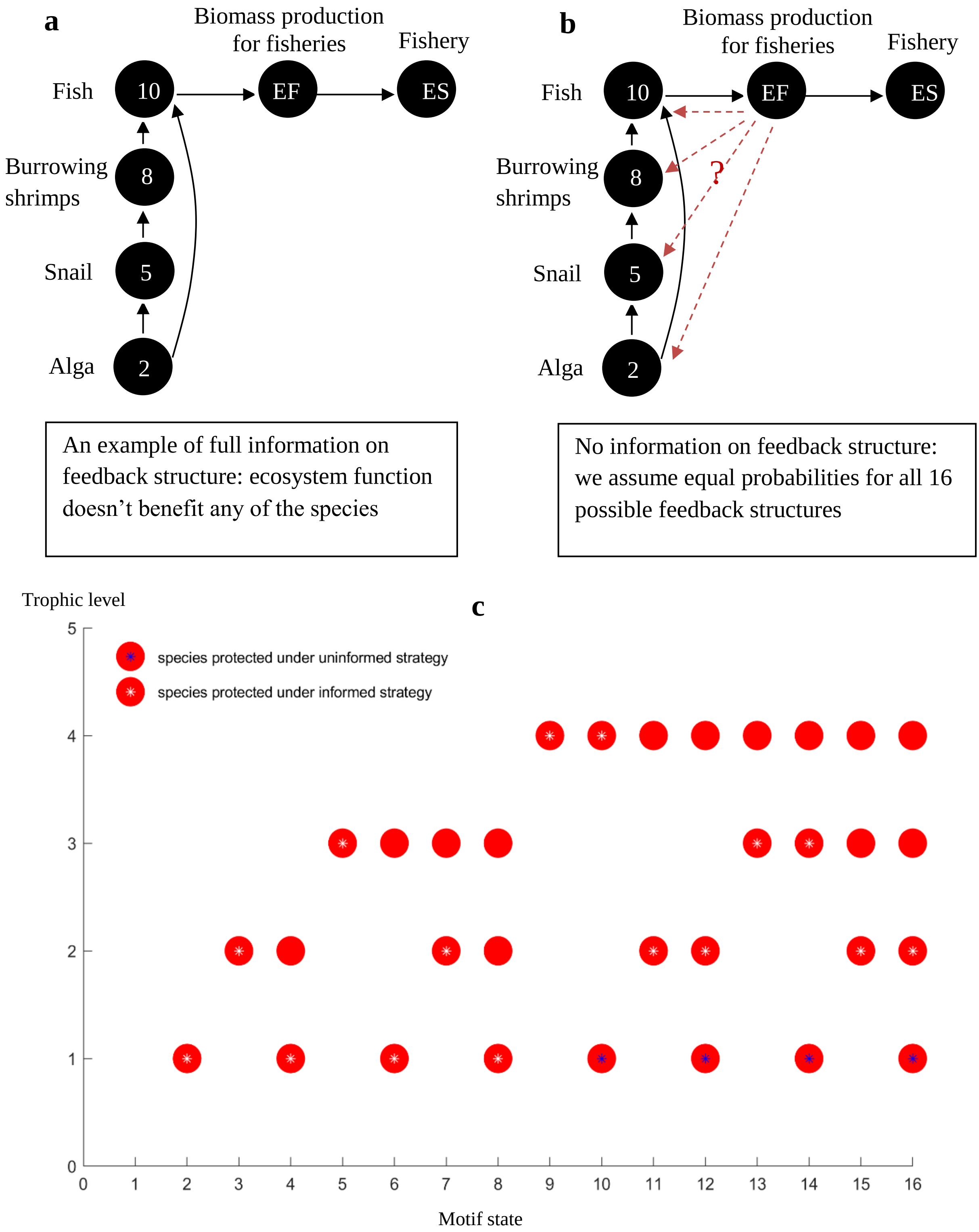
a
Biomass production for fisheries
Fishery
Fish
10
EF
ES
Burrowing shrimps
8
Snail
5
Alga
2
An example of full information on feedback structure: ecosystem function doesn't benefit any of the species
b
Biomass production for fisheries
Fishery
Fish
10
EF
ES
Burrowing shrimps
8
?
Snail
5
Alga
2
No information on feedback structure: we assume equal probabilities for all 16 possible feedback structures
c
Trophic level
species protected under uninformed strategy
species protected under informed strategy
Motif state

Supplementary Figure 6

An example from the case study to show how species management strategy could be improved with feedback information—an omnivory motif where the top predator (fish) provides the ecosystem function (biomass production for fisheries). Subtracted motif in **a** and **b** from the salt marsh ecosystem network (Supplementary Figure 10) where algae, snails, burrowing shrimps, and fish form the omnivory motif with fish providing the ecosystem function (numbers on the nodes corresponding to the functional group ID in Supplementary Figure 10). Solid arrows are known and represent predation links between functional groups and provision links from species to ecosystem function and services. In subplot **a**, feedback information is known and represented as no feedback effects from ecosystem function to species (corresponds to the first subplot in Supplementary Figure 8). In subplot **b**, feedback information is unknown and represented as red dashed arrows from ecosystem function to species. Subplot **c** shows the comparison of the optimal strategy under full feedback information (white stars) and the optimal strategy under no feedback information (blue stars) for the ecosystem service objective (with equal management cost). In subplot **c**, y axis represents the motif states from 1 (no species present in the motif) to 16 (all species are present). X axis represents the trophic level of the species in the motif from 1 (bottom trophic level) to 4 (top trophic level). For motif states where only white stars appear, the optimal informed strategy overlaps the optimal uninformed strategy.

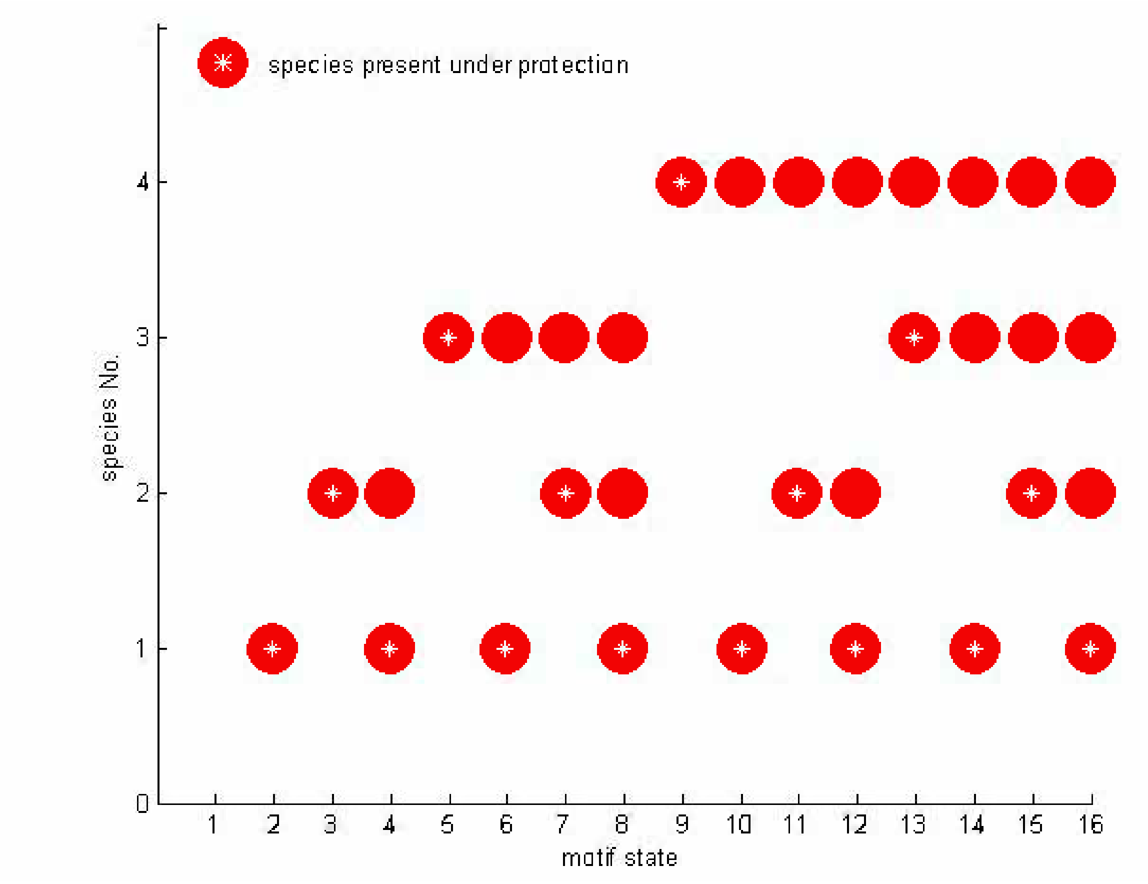

Supplementary Figure 7

Biodiversity focused strategy for the omnivory motif and the species of the third trophic level provides ecosystem function. (feedback strength=0.8, baseline survival probability=0.9, predation strength=0.9). For 16 possible feedback structures, there is a unique optimal strategy (node with white star) regardless of the feedback information. 16 possible motif states are in X axis (from initial state x=16 where all species are present to the last state x=1 all extinction). Species number 1-4 are in Y axis. Red dots indicate species presence, and red dots with white stars mean that species are under protection with Bio strategy.

When ecosystem management targets at biodiversity maximisation, the reward function of Markov Decision Process is the total number of species per food web state. Therefore, feedback information will not affect the reward matrix but only the transition probability matrix through species' survival probability. Compare to management under service maximisation where both reward function and transition matrix play in choosing the optimal strategy, strategies targeting at biodiversity are less influenced by the availability of feedback information. This could be the reason why we reach the same strategy with or without feedback information.

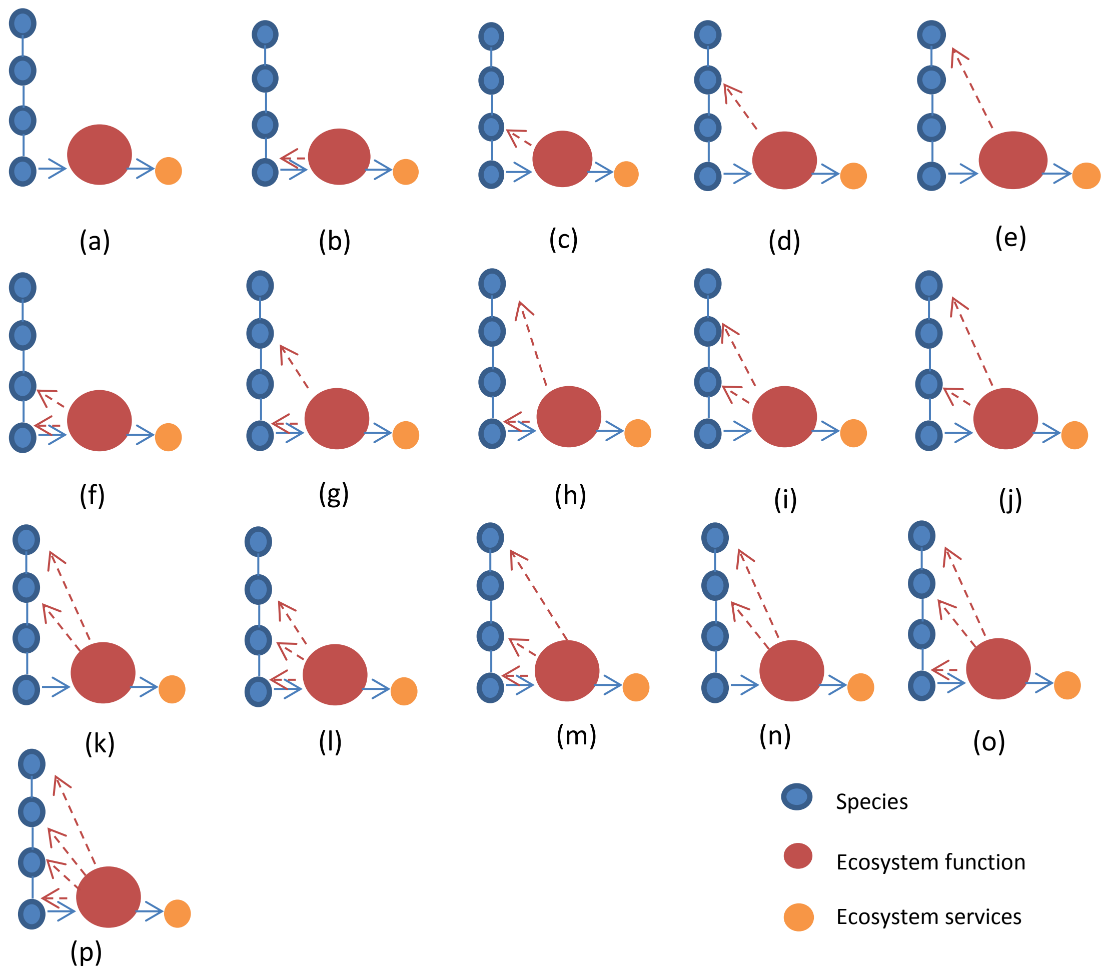

Supplementary Figure 8

Examples of the ecosystem function-species feedback structural uncertainty. For a linear motif where basal species provides the ecosystem function and services, there are 16 possible ecosystem function feedback structures. Blue, red, and orange nodes represent species, ecosystem function, and ecosystem services. The solid blue lines represent species interactions. The solid blue arrows from species nodes to ecosystem function nodes represent provisional links from species to ecosystem and from ecosystem function to services. The dotted red arrows represent unknown feedback links from ecosystem function to species. For this linear motif network, the possible feedback structures include no feedback links at all (**a**), only one feedback link from the ecosystem function to one of the four species (**b-e**), two feedback links from the ecosystem function to each two of the four species (**f-k**), three feedback links from the ecosystem function to each three of the four species (**l-o**), or ecosystem function benefits all species in the network (**p**).

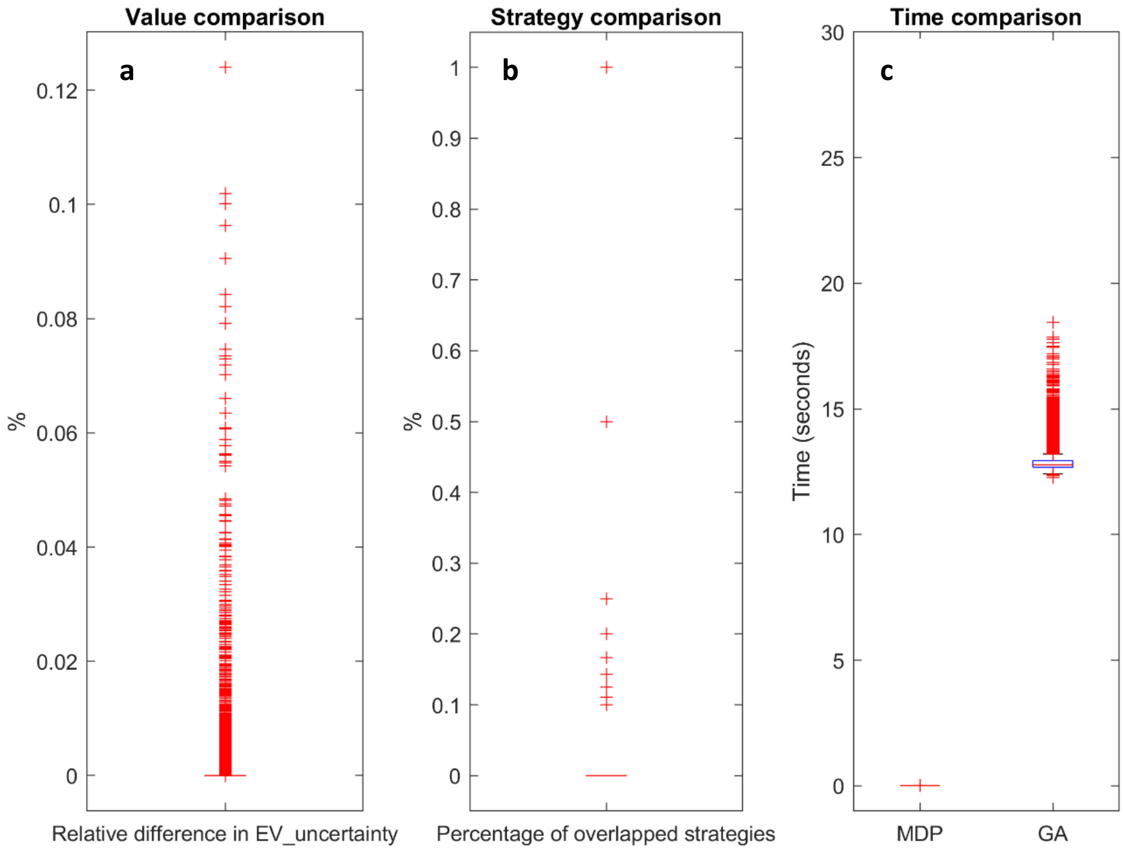

Supplementary Figure 9

A comparison between Genetic Algorithm (GA) and Markov Decision Processes (MDPs) strategies based on 10368 simulated ecosystem network. Subplot **a** represents the relative difference (in %) in expected value under uncertainty (EV_uncertainty) between MDP and GA results (obtained using EV_uncertainty of MDP strategy minus EV_uncertainty of GA strategy), where GA strategy never outperformed the optimal known structure MDP. Subplot **b** represents the percentage of overlapped strategy between GA and MDP, where we observe GA rarely reach the same strategy as MDP, indicating multiple optimal strategies exist for the same optimal value. Subplot **c** represents the time comparison between MDP and GA, where MDP takes significantly less time than GA.

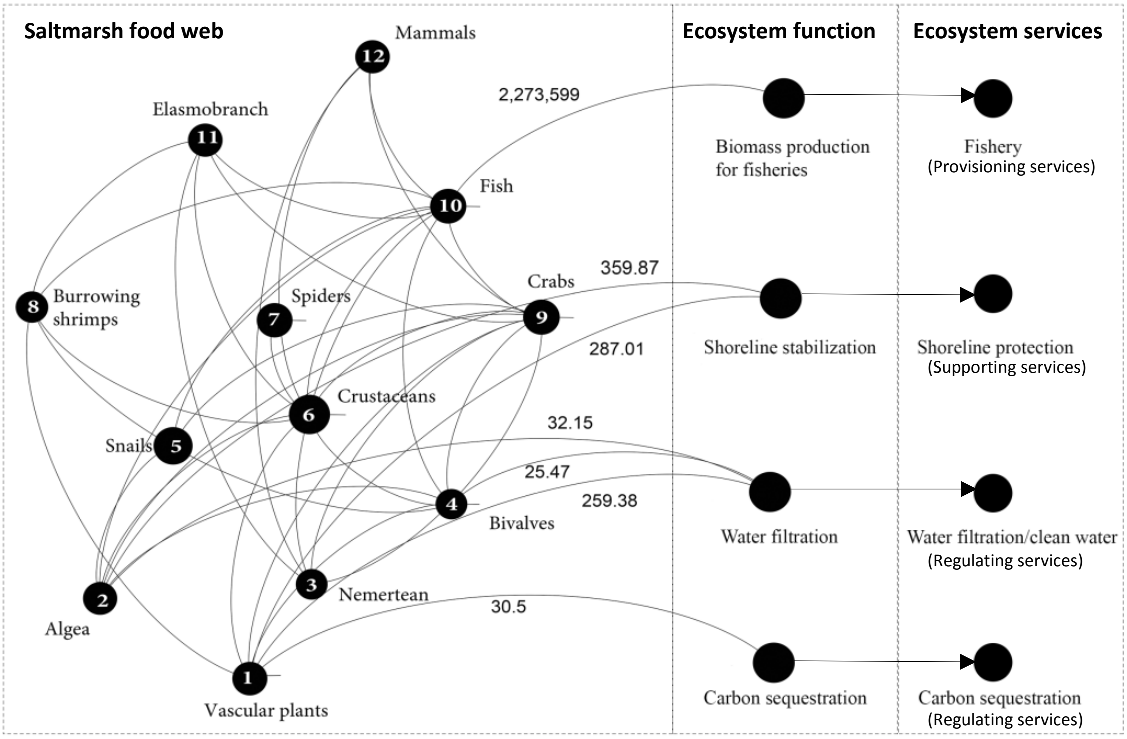

Supplementary Figure 10

Salt marsh ecosystem network for the case study. The salt marsh food web was based on Hechinger, et al. [1], where the nodes represent functional groups with group ID on it. The ecosystem function and services are identified from Xiao, et al. [2], with weighted provisional links (in US dollars) from functional groups to ecosystem functions.

Supplementary Tables

Supplementary Table 1

Result comparison between equal management cost for each species in the motif and increased management cost as the trophic level of the species increases.

Conclusion for equal management cost for each species	Results for management with increased cost as the trophic level of the species increases
1. No value of information for biodiversity objective, and high value of information for ecosystem services objective depending on the ecological features of the network (max EVPI 0.04%)	1. Consistent (Supplementary Figure 3)
2. Importance of the five ecological features*: trophic level> motif>baseline probability of survival (p_j^0)> predation strength (b) >feedback strength (α)<br><br>Overall<br>trophic 1.0327046<br>pj0 0.2657015<br>motif 0.2414745<br>b 0.2339432<br>alpha 0.2056920	2. Consistent for the most important ecological parameter (trophic level): trophic level>feedback strength (α)>baseline probability of survival (p_j^0)>motif>predation strength (b)<br><br>Overall<br>trophic 0.99336775<br>alpha 0.93140139<br>pj0 0.66265203<br>motif 0.14745632<br>b 0.08628965
3. For ecosystem services objective, no value of information when basal species provide the ecosystem function	Consistent (Supplementary Figure 4).
4. Ecological configuration for the highest relative EVPI (25%): omnivory motif, top predator provides the ecosystem function, high baseline probability of survival, high feedback strength, and high predation strength	Consistent. The same ecological configuration reaches the highest relative EVPI: 72%. (Supplementary Figure 3, Figure 4)

*The parameter importance is calculated using the decision tree analysis in R (*rpart* with anova method, complexity parameter=0.02, maxdepth=7, and minsplit=20)

Supplementary Methods

Supplementary Methods 1. Minimal expected regret and min-max regret approaches

The EVPI relies on the computation of an expected difference, and for this reason it is a risk neutral criterion. Indeed, let us define the more general quantity $EVPI(\delta)$ for a given strategy δ as

$$EVPI(\delta) = \frac{1}{F}\sum_{f=1}^{F} V_{\delta_f^*}(x^0, f) - \frac{1}{F}\sum_{f=1}^{F} V_\delta(x^0, f) \quad (1)$$

$$= \frac{1}{F}\sum_{f=1}^{F}(V_{\delta_f^*}(x^0, f) - V_\delta(x^0, f)) = \frac{1}{F}\sum_{f=1}^{F} R(\delta, f) \quad (2)$$

Where $R\ (\delta, f) = \ (V_{\delta_f^*}(x^0, f) - V_\delta(x^0, f)$ is called the regret. Expression $EVPI(\delta)$ is equal to the expected regret of a decision-maker choosing strategy δ when "Nature" chooses uniformly at random the feedback structure f, instead of choosing the true unknown optimal strategy. Then by definition,

$$EVPI = EVPI(\delta^*) = \min_\delta EVPI(\delta) \quad (3)$$

So δ^* which minimizes *EVPI*[3] minimizes the expected regret.

Instead of minimizing the expected regret, which makes the (unreasonable) assumption that every feedback structures are equal probable (or, more generally, that their distribution is known), we can choose to minimize the larger regret that could be reached. For this, the minimax regret criterion [4] can be used. Indeed, this criterion makes no assumption about a feedback structure distribution and is actually "cautious": it amounts to choosing the strategy which minimizes regret when Nature is assumed to be malevolent, i.e. when Nature chooses the worst possible structure, knowing the strategy we have chosen. In other terms, the maximum regret expression of strategy δ is:

$$MR(\delta) = \max_f(V_{\delta_f^*}(x^0, f) - V_\delta(x^0, f)) = \max_f R(\delta, f) \quad (4)$$

And the minimax regret strategy is the strategy δ^* which minimizes $MR(\delta)$.

Note that, for any strategy δ,

$$EVPI(\delta) = \frac{1}{F}\sum_{f=1}^{F} VPI(\delta, f) \leq \max_f R(\delta, f) = MR(\delta) \quad (5)$$

Thus, $EVPI$ and $VPI_{high} = \min_{\delta} \max_{f} R(\delta, f)$ respectively correspond to the minimum expected regret and minimax regret one incurs when not knowing the true feedback structure. In other terms, both quantities can be used as measures of the (expected or maximum) value of perfect information of the feedback structure.

Supplementary Methods 2. Using Markov Decision Processes to model species dynamics and protection actions effects

Equations (2), (3) and (4) in the main text remain very general. To apply them to the dynamics of an ecosystem, we must define a model for the species dynamics and for the effect of protecting a species on the survival of all species. We propose a Markov Decision Process model, since this will enable us to exploit the associated algorithmic tools to compute optimal strategies.

A MDP model is defined by five elements: the ecosystem state space **X**, the action space **A**, the probability transition matrix **P**, the reward function **R**, and the time horizon **T**.

We defined the ecosystem state space **X** as the combination of individual species state, where each species states space is {0,1}. Let x_j in {0,1} be the state of species $j \in \{1, \dots, J\}$ at any given time. If species *j* is present at time t, $x_j = 1$, and if it is absent $x_j = 0$. For four species, there are 2^4=16 possible ecosystem states made of every possible combination of species' state.

Let a in **A = { 1, …, J}** be the species that a decision maker chooses to protect at a given time, i.e. $a = j$ indicates that species j is chosen for protection.

Markov Decision Processes assume that the state at time *t+1* only depends on the state and the action at time *t*. Let ***P*** be the transition probability matrix representing the dynamics of the system from time step *t* to time step *t+1*. $P(x^{t+1} | x^t, a^t, \{p_j^0\}_j, \alpha, b, f, M)$ represents the conditional probability of the ecosystem transitioning from state x^t to x^{t+1} given action a^t is implemented at time *t*. This transition probability is also conditional on the baseline probability of survival of species *j*— p_j^0, the feedback strength α (the percentage of the ecosystem function going back to a species), the predation strength b, the feedback structure f and the food web matrix M representing the prey-predator interactions of our system. To model this transition probability, we assumed that, knowing the state at time t, x^t , the

state of species *j* at time *t+1* is independent of the state of the other species at time *t+1*. So we can define the transition probability P as the product of J individual species' transition probabilities:

$$P\left(x^{t+1}\middle|x^t, a^t,\ \mathrm{p_j^0}, \alpha, b, f, M\right) = \prod_{j=1}^{J} P_j\left(x_j^{t+1}\middle|x^t\ , a^t,\ \mathrm{p_j^0}, \alpha, b, f, M\right) \quad (6)$$

$P_j\left(x_j^{t+1}|x^t\ = 1\ , a^t,\ \mathrm{p_j^0},\ \alpha, b, f, M\right)$ represents the probability of a species becoming absent ($x_j^{t+1} = 0$) or remaining present ($x_j^{t+1} = 1$). Accounting for top-down effects, we assumed that this probability should depend on the prey neighbourhood size $\mathrm{N_{prey}}(j, x^t, M)$ i.e. the number of extant prey of species *j* in state x^t, the predator neighbourhood size $\mathrm{N_{predator}}(j, x^t, M)$ i.e. the number of extant predator of species *j* in x^t, and $\mathrm{N_{EF}}(j, x^t, f, \alpha)$ i.e. the amount of ecosystem function received by species *j* if the ecosystem is in state x^t, for a given feedback structure f and a feedback strength α. Survival probability of a species will increase with the number of extant preys $\mathrm{N_{prey}}(j, x^t, M)$ and ecosystem function available $\mathrm{N_{EF}}\ (j, x^t, f)$, and will decrease with the number of extant predators $\mathrm{N_{predator}}(j, x^t, M)$. We assumed that $\mathrm{N_{prey}}(j, x^t, M)$, $\mathrm{N_{EF}}(j, x^t, f)$, and $\mathrm{N_{predator}}(j, x^t, M)$ are maximum at the initial time step where all species are present (i.e. $x^t = x^0 =\ [1,1,1,1]$). Formally, we defined the transition probability when species *j* is not under protection ($a^t \neq j$) as the product of four terms:

$$P_j^t\left(x_j^{t+1} = 1\middle|x_j^t = 1, a^t \neq j, \mathrm{p_j^0}, \alpha, b, f, M\right) = p_j^0 * \frac{\mathrm{N_{prey}}(j,x^t,M)}{\mathrm{N_{prey}}(j,x^0,M)} * \left(1 - b\,\frac{\mathrm{N_{predator}}(j,x^t,M)}{\mathrm{N_{predator}}(j,x^0,M)}\right) * \frac{\mathrm{N_{EF}}(j,x^t,f,\alpha)}{\mathrm{N_{EF}^*}(j,f)} \quad (7)$$

The first term, p_j^0, represents the baseline probability of survival for species *j*. We assumed that p_j^0 decreases as the trophic level of a species *j* increases [5] using the fractional trophic level formula [6]. The second term, $\frac{\mathrm{N_{prey}}(j,x^t,M)}{\mathrm{N_{prey}}(j,x^0,M)}$, is the ratio of the current number of extant preys over the initial number of prey available for species j. The third term, $\left(1 - b\,\frac{\mathrm{N_{predator}}(\mathrm{j},\mathrm{x^t},M)}{\mathrm{N_{predator}}(\mathrm{j},\mathrm{x^0},M)}\right)$, represents the predators' influence; it starts from one minus a predation strength *b* (*b* is lower than 1) at initial state where all predators are present (i.e. $\mathrm{N_{predator}}(j, x^t, M) =\ \mathrm{N_{predator}}(j, x^0, M)$), and increases towards 1 as predators go extinct. The fourth term, $\frac{\mathrm{N_{EF}}(j,x^t,f,\alpha)}{\mathrm{N_{EF}^*}(j,f)}$, represents the ecosystem function benefit to species *j*. It is equal to 1 in absence of feedback links (Supplementary Figure 8**a**) or, in presence of feedback links (Supplementary Figure 8(**b-p**) it is defined as the current amount of ecosystem function received $\mathrm{N_{EF}}(j, x^t, f, \alpha)$ over the maximum amount of ecosystem function required by species j, $\mathrm{N_{EF}^*}(j, f)$. The current amount of ecosystem function is calculated as $\mathrm{N_{EF}}(j, x^t, f, \alpha) = \frac{\alpha*100}{J^t(x^t,f)}$, where we assumed a total amount of 100 unit of ecosystem function is provided for a given

ecosystem. $J^t(x^t, f)$ represents the number of extant species at time *t*, which depends on the current ecosystem state x^t and the feedback structure f. For simplicity, we assumed that the maximum amount of ecosystem function required by species *j* is obtained at initial state with 80% of the total ecosystem function going back to species, and is feedback structure dependent, i.e. $\mathrm{N}^*_{\mathrm{EF}}(j, f) = 0.8\frac{100}{J^t(f)}$. Thus, the fourth term is equal to $\frac{\alpha}{0.8} * \frac{J^t(f)}{J^t(x^t,f)}$.

In this way, under the most favourable condition where species *j* has no predator, no prey loss and receive maximum level of ecosystem function, Supplementary Equation 7 reduces to its baseline probability of survival p_j^0. However, species *j* survival probability will decrease when at least one of the following three events happen—prey loss, predator presence, or insufficient functional support. We also assumed no recolonization is possible, i.e. a species will not be present at *t*+1 if it was absent at the previous time step.

The reward function R measures the ecosystem outcomes given an ecosystem state $x^t$and an action a^t. We assumed equal management cost for each species (also tested higher management cost with higher trophic levels, please see below details). Here, we considered two different management objectives: biodiversity and ecosystem services. For the biodiversity objective, $\mathrm{R}(x^t, a^t)$ is the total number of species extant in the food web minus the management cost a^t, where a^t specifies the species to protect at time *t*. For the ecosystem services objective, $\mathrm{R}(x^t,\ a^t)$ is the total value (US dollars) of services provided by the system minus the management cost a^t:

$$R_{Bio}(x^t) = \sum_{j=1}^{4} x_j^t - cost(a^t) \quad (8)$$

$$R_{ES}(x^t) = \sum_{j=1}^{4} ES(x_j^t) - cost(a^t) \quad (9)$$

We assumed an infinite time horizon ***T*** in this decision-making process.

Supplementary Methods 3. Value of a strategy, δ

The quality of δ can be measured by its value function. This is defined, for all possible initial states of the system, as the discounted expected sum of rewards over an infinite time horizon:

$$\mathrm{V}_\delta\,(x^1) = \mathrm{E}[\sum_{t=1}^{T\infty} \gamma^{\mathrm{t}} R(x^t)\ \mid \delta, x^1] \quad (10)$$

Where $\gamma\ \epsilon$] 0, 1 [is a discount factor ($\gamma = 0.96$ in the study).

Therefore, an optimal strategy δ_f^*, also called the informed strategy, gives the maximum expected value of ecosystem outcomes when the feedback structure is f among all strategies δ, for every

possible starting states x^0. In particular, assuming the starting point is the initial state x^0 where all species are present:

$$V_{\delta_f^*}(x^0, f) \geq V_\delta(x^0, f),\ where\ x^0 = [1\ 1\ 1\ 1] \qquad (11)$$

Supplementary Methods 4. Genetic Algorithm approximation

Because our calculations of the EV_uncertainty using a finite set of models may not provide the exact values, we also apply a genetic algorithm approach [7]. The first step is to randomly select 1000 samples from 4^{16} strategies as the 'parent' and calculate the corresponding value functions using those sample strategies. We then compare those value functions with an initial value (e.g. zero), and update the value function if a larger one appears. In the end, we obtain the optimal strategy with the maximum value function from this parent sample. The second step is to build 'child' sample and reproducing. We randomly choose two strategies from the parent sample and compare, the one with larger value function goes to the child sample. Then we apply bit string mutation to replace that strategy with a 'new strategy'. This process is repeated 1000 times and the child sample will contain exactly the same number of strategies (but not necessary better) as the parent sample. The purpose of building child sample using random comparison and not based on the maximum value from parent sample is to avoid local optimal values. Finally, the last step is to update 'parent' with the obtained 'child', and then start from the first step again. Therefore, we continue to update the maximum value function and optimal strategy 1000 times to reach the convergence.

We used MDP certainty solutions to approximate the EV_uncertainty based on the following considerations:

1. Finding the optimal solution with uncertain problem structure is hard, because it is no longer a MDP but rather an "hidden model MDP" [8] where there are 16 different possible MDPs. Chades, et al. [8] have shown that solving optimally this problem has a high computational complexity (in terms of computational complexity theory, it is PSPACE-hard to solve). Thus, it is unlikely that there exist an exact optimisation method which solves the problem significantly faster than by evaluating the entire solution space (all "4-billions+" strategies) over the 16 possible feedback network structures.
2. The MDP community has considered problems with model structure uncertainty. Apart from the Partially observable MDP inspired methods [8,9], active learning methods [10] or model-based Bayesian reinforcement learning approaches [11] could be applied for solving our problem. However, these approaches do not come with the guarantee of finding an optimal strategy in reasonable time. We chose instead to apply a heuristic solution method. This consists in looking for a strategy which solves optimally at least one problem with known feedback structure. This is reasonable since (i) these strategies are in reasonable number; (ii) each of

these strategies solves optimally at least one potentially existing problem, unlike uncertainty optimal strategies which solve the problem "in expectation" (therefore might not corresponds to a real problem).

3. Applying genetic algorithms is an empirical way of comparing our MDP solutions that explores 16 solutions with an approach that explores 20,000 solutions at a significant computational cost. We observed that genetic algorithms most often (85% of problems) converged to the optimal known structure MDP strategy of the same value). The genetic algorithms strategy never outperformed the optimal known structure MDP, which strongly suggests that, for the considered problems, our approximation is very good (Supplementary Figure 9).

Supplementary Methods 5. Salt marsh case study

To test the utility of our theoretical approach using a real ecosystem, we identify four different motifs from functional groups within the salt marsh food web from Hechinger, et al. [1], with associated ecosystem functions from Xiao, et al. [2] (Supplementary Figure 8). The linear motif includes vascular plants, bivalves, crustaceans, and fish. The apparent competition motif includes vascular plants, burrowing shrimps, crabs, and fish. The omnivory motif includes algae, snails, burrowing shrimps, and fish. The intraguild competition motif includes algae, bivalves, crustaceans, and fish. The salt marsh case study also provides information on species providing several ecosystem functions: vascular plants provide shoreline stabilization; bivalves and algae provide water filtration; and fish provide biomass production for fishery (see Figure 4 in the main text).

To further explain the process of calculating EVPI for specific motifs in the case study, we use the omnivory motif where the top trophic level (fish) provide the ecosystem function (biomass production for fisheries) as an example. With this ecosystem network structure, we follow the same steps as the theoretical approach to calculate the relative EVPI (see main steps in Supplementary Figure 1), with the same assumptions such as exponentially decreased baseline survival probability as the trophic level of the species increases, equal management cost for species protection, no rewiring of the network, and a maximum of 80% of an ecosystem function going back to support the species' survival. Due to the lack of information on the feedback strengths (α), baseline probabilities of survival (p_j^0), and predation strength (b), we calculate the relative EVPI for all values tested in our theoretical approach (see Table 1 in the main text). We then take the maximum of the relative EVPI (EVPI=25%) which occurs when 80% of function going back to species, 0.8 baseline probability of survival, and 0.9 predation strength (see[12] for Matlab code for the case study).

Supplementary Discussion

Omnivory motif had the highest relative EVPI in the case study

We found that the ecosystem configuration with the maximum relative EVPI (25.44%) from the case study is consistent with the theoretical findings—i.e., omnivory motif (algae, snails, burrowing shrimps, and fish) with a top predator (fish) providing the ecosystem function (Fig. 4). If there is a positive feedback from fish biomass production to lower trophic level functional groups, it could be the indirect top-down effects that were not captured in the prey-predator relationship in the model. For example, having more fish biomass could negatively influence the survival of shrimps (one trophic level lower through direct predation) but could positively influence the survival of algae (two trophic level lower through the indirect effect). In Supplementary Equation 7, the direct effect is captured by the term $\frac{N_{prey}(j,x^t,M)}{N_{prey}(j,x^0,M)}\left(1 - b\frac{N_{predator}(j,x^t,M)}{N_{predator}(j,x^0,M)}\right)$ and the indirect effect could be captured as the ecosystem function-species feedback effect by the term $\frac{N_{EF}(j,x^t,f,\alpha)}{N^*_{EF}(j,f)}$. Therefore, at the same time step t, fish could directly influence its prey and indirectly influence the bottom species algae if there is a feedback link from fish biomass to algae. However, beyond direct effects through trophic dynamics for lower trophic levels, there is currently uncertainty over if other positive feedbacks from fish biomass to lower trophic levels exist. The focus of our study is to calculate the value of having information about the feedback in terms of management outcome improvement, if positive feedback links exist, in the form of indirect effect or other non-trophic relationship (e.g. mutualism). We find that the management strategy and outcomes could be greatly improved by collecting the information on whether or not there exist other positive but currently unknown feedbacks from biomass production for fishery to lower trophic level functional groups. We also consider, as one of the 16 possible feedback structures, that the possibility that no additional positive feedbacks exists (i.e., Supplementary Figure 6**a**). Supplementary Figure 6**c** shows the strategy difference between the optimal management strategy with full information on the feedback structure (Supplementary Figure 6**a**) and the optimal management strategy facing uncertainty (equal probability for each of the 16 possible feedback structures, Supplementary Figure 6**b**).

Supplementary References

1 Hechinger, R. F. *et al.* Food webs including parasites, biomass, body sizes, and life stages for three California/Baja California estuaries: Ecological Archives E092-066. *Ecology* **92**, 791-791 (2011).

2 Xiao, H. *et al.* Win‐wins for biodiversity and ecosystem service conservation depend on the trophic levels of the species providing services. *Journal of applied ecology* **55**, 2160-2170 (2018).

3 Nations, F. a. A. O. o. t. U. trophic levels. doi:http://www.fao.org/fishery/topic/4210/en (2018).

4 Savage, L. J. The theory of statistical decision. *Journal of the American Statistical association* **46**, 55-67 (1951).

5 Purvis, A., Gittleman, J. L., Cowlishaw, G. & Mace, G. M. Predicting extinction risk in declining species. *Proceedings of the Royal Society of London. Series B: Biological Sciences* **267**, 1947-1952 (2000).

6 Odum, W. E. & Heald, E. J. The detritus-based food web of an estuarine mangrove community. *Estuarine Research: Chemistry, Biology, and the Estuarine System.* **1**, 265-286 (1975).

7 Davis, L. Handbook of genetic algorithms. (1991).

8 Chades, I. *et al.* in *Twenty-Sixth AAAI Conference on Artificial Intelligence.*

9 Ong, S. C., Png, S. W., Hsu, D. & Lee, W. S. Planning under uncertainty for robotic tasks with mixed observability. *The International Journal of Robotics Research* **29**, 1053-1068 (2010).

10 Araya-López, M., Buffet, O., Thomas, V. & Charpillet, F. in *European Workshop on Reinforcement Learning.* 42-53 (Springer).

11 Ross, S. & Pineau, J. in *Uncertainty in artificial intelligence: proceedings of the... conference. Conference on Uncertainty in Artificial Intelligence.* 476 (NIH Public Access).

12 Xiao, H. VOI paper equal cost. figshare. Fileset., doi:https://doi.org/10.6084/m9.figshare.7712090.v1 (2019).
