# Supplementary material for: The value of understanding feedbacks from ecosystem functions to species for managing ecosystems
Source: Nat Commun. 2019 Aug 29;10:3901. doi: 10.1038/s41467-019-11890-7 (PMC6715698; doi:10.1038/s41467-019-11890-7)
Supplement: Supplementary file 2 — Reporting Summary [file 41467_2019_11890_MOESM2_ESM.pdf]

nature research

Corresponding author(s): Hui Xiao

Last updated by author(s): 29-06-2019

Reporting Summary

Nature Research wishes to improve the reproducibility of the work that we publish. This form provides structure for consistency and transparency in reporting. For further information on Nature Research policies, see Authors & Referees and the Editorial Policy Checklist.

Statistics

For all statistical analyses, confirm that the following items are present in the figure legend, table legend, main text, or Methods section.

n/a	Confirmed	
☐	☒	The exact sample size (*n*) for each experimental group/condition, given as a discrete number and unit of measurement
☒	☐	A statement on whether measurements were taken from distinct samples or whether the same sample was measured repeatedly
☒	☐	The statistical test(s) used AND whether they are one- or two-sided<br>*Only common tests should be described solely by name; describe more complex techniques in the Methods section.*
☐	☒	A description of all covariates tested
☐	☒	A description of any assumptions or corrections, such as tests of normality and adjustment for multiple comparisons
☒	☐	A full description of the statistical parameters including central tendency (e.g. means) or other basic estimates (e.g. regression coefficient) AND variation (e.g. standard deviation) or associated estimates of uncertainty (e.g. confidence intervals)
☒	☐	For null hypothesis testing, the test statistic (e.g. *F*, *t*, *r*) with confidence intervals, effect sizes, degrees of freedom and *P* value noted<br>*Give P values as exact values whenever suitable.*
☒	☐	For Bayesian analysis, information on the choice of priors and Markov chain Monte Carlo settings
☒	☐	For hierarchical and complex designs, identification of the appropriate level for tests and full reporting of outcomes
☒	☐	Estimates of effect sizes (e.g. Cohen's *d*, Pearson's *r*), indicating how they were calculated

Our web collection on statistics for biologists contains articles on many of the points above.

Software and code

Policy information about availability of computer code

Data collection	We used simulations and empirical salt marsh food web data from published literatures (Xiao, et al. (2018) and Hechinger, et al. (2011)).
Data analysis	Major analysis including ecosystem network design, optimization, Markov Chain were conducted using Matlab version R2017b.We conducted the optimization using Markov Decision Processes toolbox in Matlab (version R2017b) developed by Chades et al (2014). The decision tree analysis and data management were conducted using R studio.

For manuscripts utilizing custom algorithms or software that are central to the research but not yet described in published literature, software must be made available to editors/reviewers. We strongly encourage code deposition in a community repository (e.g. GitHub). See the Nature Research guidelines for submitting code & software for further information.

Data

Policy information about availability of data

All manuscripts must include a data availability statement. This statement should provide the following information, where applicable:

- Accession codes, unique identifiers, or web links for publicly available datasets
- A list of figures that have associated raw data
- A description of any restrictions on data availability

Our Matlab code to reproduce all simulations is available via Figshare:
https://doi.org/10.6084/m9.figshare.6668087.v1
https://doi.org/10.6084/m9.figshare.7712090.v1

Field-specific reporting

Please select the one below that is the best fit for your research. If you are not sure, read the appropriate sections before making your selection.

☐ Life sciences ☐ Behavioural & social sciences ☒ Ecological, evolutionary & environmental sciences

For a reference copy of the document with all sections, see nature.com/documents/nr-reporting-summary-flat.pdf

Ecological, evolutionary & environmental sciences study design

All studies must disclose on these points even when the disclosure is negative.

Study description	We simulate network motifs to capture the species interactions and we model the ecosystem dynamics on a network motif, the ecosystem function and the services. We assume that without management, each species has a probability of extinction at each time step and managers must decide which species to manage. We do not know which species benefit from the ecosystem function, and we calculate the Expected Value of Perfect Information (EVPI) to determine the value of resolving this uncertainty. Then, we investigate how EVPI changes across five ecological features: different motifs, trophic level of the function, feedback strengths (α), baseline probabilities of survival (p_j^0), and predation strength(b). In addition, we compare the ecological features of the maximum EVPI, also called the 'expected regret', with those features of the 'maximum regret' that a manager could have when the feedback information is not available. To illustrate the value of our work to real-world situations, we apply our approach to an empirical salt marsh ecosystem in Carpinteria Salt Marsh Reserve, CA, USA from Xiao, et al. and Hechinger, et al..
Research sample	NA
Sampling strategy	NA
Data collection	We use the empirical salt marsh ecosystem data from Xiao, et al. and Hechinger, et al..
Timing and spatial scale	NA
Data exclusions	NA
Reproducibility	Results are reproducible and we provide the Matlab and R code via fishare.
Randomization	NA
Blinding	NA

Did the study involve field work? ☐ Yes ☒ No

Reporting for specific materials, systems and methods

We require information from authors about some types of materials, experimental systems and methods used in many studies. Here, indicate whether each material, system or method listed is relevant to your study. If you are not sure if a list item applies to your research, read the appropriate section before selecting a response.

Materials & experimental systems

n/a	Involved in the study	
☒	☐	Antibodies
☒	☐	Eukaryotic cell lines
☒	☐	Palaeontology
☒	☐	Animals and other organisms
☒	☐	Human research participants
☒	☐	Clinical data

Methods

n/a	Involved in the study	
☒	☐	ChIP-seq
☒	☐	Flow cytometry
☒	☐	MRI-based neuroimaging
